# A specific role for endothelial EPLIN-isoform-regulated actin dynamics in neutrophil transmigration

**DOI:** 10.1038/s41598-025-98192-9

**Published:** 2025-05-05

**Authors:** Mohammed Aldirawi, Parisa Ghanbari, Magdalena Mietkowska, Sigrid März, Maria Odenthal-Schnittler, Jonas Franz, Julian Wegner, Silke Currie, Jan Philip Kipcke, Muna Taha, Marcus Giglmaier, Anja Blanque, Hermann Schillers, Erez Raz, Dietmar Vestweber, Klemens Rottner, Hans Schnittler

**Affiliations:** 1 Institute of Anatomy and Vascular Biology, University Münster, Vesaliusweg 2-4, Münster, Germany; 2 Max Planck Institute for Molecular Biomedicine, Röntgenstraße 20, Münster, Germany; 3 Division of Molecular Cell Biology, Zoological Institute, Technische Universität Braunschweig, Spielmannstrasse 7, 38106 Braunschweig, Germany; 4 Molecular Cell Biology Group, Helmholtz Centre for Infection Research, Inhoffenstrasse 7, 38124 Braunschweig, Germany; 5 Institute of Neuropathology, University of Münster, Pottkamp 2, 48149 Münster, Germany; 6 Institute of Cell Biology, Center for Molecular Biology of Inflammation, 48149 Münster, Germany; 7 Institute of Aerodynamics and Fluid Mechanics, Technical University of Munich, Boltzmannstr. 15, 85748 Garching, Germany; 8 Institute of Physiology, University Münster, Robert-Koch Strasse 27a, 48149 Münster, Germany

**Keywords:** Transendothelial migration, Cell junction dynamics, Arp2/3 complex, Lamellipodia, Stress fibres, VL, JAIL, Membrane stiffness, Actin binding proteins, VE-cadherin, Cell signalling, Inflammation

## Abstract

Proinflammatory cytokines such as TNF-α or IL-1β activate the endothelium promoting leukocyte transendothelial migration (TEM) via expression of cell adhesion molecules (CAM) and cause actin remodelling. However, the function of endothelial actin remodelling in TEM remains elusive, despite its involvement in the formation of docking structures, diapedesis pores and pore resealing. Here, we establish EPLIN-isoforms, EPLIN-β and EPLIN-α, as differential regulators of TNF-α-inducedactin-remodelling significantly affecting TEM. We find EPLIN-β-induced stress fiber formation upon TNF-α-treatment weakens endothelial junctions, upregulates junctional dynamics and facilitates intercellular gaps for TEM. Increased junctional dynamics involves branched actin filaments under the control of EPLIN-α, including docking structure formation and transmigratory pore closure. We further establish by EPLIN deletion and re-expression studies that EPLIN-α-mediated termination of branched actin filaments maintains TNF-α-induced junctional dynamics and intercellular gaps facilitating TEM. These findings highlight the critical role of TNF-α-induced differential actin dynamics, controlled by EPLIN isoforms, in TEM. These results also offer a wider understanding of inflammation-induced TEM by incorporating altered junctional dynamics alongside upregulation of cell adhesion molecules.

## Introduction

Extravasation of leukocytes through endothelial cells (ECs) is essential in immune response after trauma and inflammation. This process requires endothelial activation by pro-inflamatory mediators such as TNF-α which is released by activated immune cells^1^. TNF-α leads to the expression of cell adhesion molecules (CAM) including selectins, ICAM-1 and VCAM^2,3^, which in turn bind to leukocyte ligands such as β2-integrins, LFA-1 (αLβ2), Mac-1 (αMβ2), as well as β1-integrin and VLA-4 (α4β1) (reviewed in^4–6^). These interactions lead to a sequence of events referred to as margination/capture, rolling, firm adhesion, spreading and crawling to endothelial exit sites including hotspots and finally transmigration^7–11^. TNF-α is also known to alter endothelial actin distribution by inducing stress fibres and increasing junctional dynamics^12–14^. Although there is a consensus that actin affects transendothelial migration (TEM), the underlying mechanisms, particularly with respect to different types of actin filaments such as stress fibres and Arp2/3 complex controlled actin filaments in the endothelium^15^, remains elusive. Initiation of TEM of neutrophils depends on LFA-1-induced ICAM-1 clustering on the endothelial surface, which recruits endothelial actin to the transmigration site^5,16–18^ forming an actin ring-like structure around transmigrating leukocytes^19,20^. This process is also described to be associated with membrane protrusions such as ‘docking structures’ or ‘transmigratory cups’^21–24^. Other reports, however, challenged this concept as they have observed actin rings, but no membrane protrusions or filopodia^25–27^. Irrespective of these unsolved issues, there is a consensus that TEM occurs through micrometre-sized pores in or between endothelial cells^5,28–30^. The role of actin filaments in the endothelium in the actual TEM process is incompletly understood. However, after TEM, the closure of endothelial pores is mediated by the formation of actin-driven membrane protrusions called ventral lamellipodia accompanying the restoration of VE-cadherin adhesion^24,31–33^. As opposed to the expression of cell adhesion molecules, less attention is usually paid to other effects in the endothelium induced by TNF-α activation. This includes actin remodelling characterized by stress fiber formation and increased membrane protrusion dynamics, accompanied by increased permeability^14,34–36^.

Endothelial cells posses a complex actin cytoskeleton that is essential for structural integrity, migration, response to stimuli and vascular function^15,18,37–39^. Controlled by actin-binding proteins and Rho GTPases^40,41^, it comprises linear actin bundles (forming junctional actin and stress fibres) and branched actin networks. The latter, regulated by the WASP/WAVE/Arp2/3 complex^42–44^, drive lamellipodia and membrane protrusions essential for cellular remodelling^1^, although the Arp2/3 complex can also influence linear filopodia formation through SPIN90/Dip proteins (for review see^45^).

Here we aim to analyse the specific contribution of TNF-α-induced stress fibres and Arp2/3 complex-dependent actin filaments to TEM through the control of the actin-binding epithelial protein lost in neoplasm (EPLIN) isoforms, in particular EPLIN-α and EPLIN-β. Both stress fibres and branched actin filament-driven membrane protrusions are the major components of the endothelial actin cytoskeleton and exert different functions in the endothelium^15^. Endothelial stress fibres are contractile and play an important role in initiation of endothelial permeability and equally contribute to determining endothelial biomechanical properties^46–51^, parameters supposed to be important for leukocyte TEM as well. Similarly, branched actin networks in protrusions are supposed to contribute to actual TEM and pore closure^37,39^. Previous studies demonstrated that those protrusions are critical for maintenance of overall monolayer integrity and cell motility. The described protrusions include classical lamellipodia for single-cell migration^52,53^, as well as cell junction-associated protrusions in endothelial monolayers, termed ventral lamellipodia (VL), lateral lamellipodia, junction-based lamellipodia, or junction-associated intermittent lamellipodia (JAIL)^32,33,54–59^. Among these protrusions, JAILs are defined as small, multiple and repeatedly appearing junctional protrusions that directly restore weakened or lost VE-cadherin adhesions, thereby ensuring the integrity of the endothelial monolayer at quiescent conditions and during cellular remodelling^60^.

To investigate the influence of endothelial stress fibres *versus* branched actin filaments during leukocyte TEM, we study the actin-binding epithelial protein lost in neoplasm (EPLIN) isoforms^61–64^. These studies build on our previous work showing that EPLIN-β preferentially labels and stabilises actin bundles like stress fibres, whereas EPLIN-α preferentially labels branched actin-driven membrane protrusions. In addition, EPLIN-α functions to terminate the extension of Arp2/3 complex-dependent branched actin filament networks at endothelial cell junctions, thereby regulating the dynamics of membrane protrusions. In this scenario, EPLIN-α-controlled mechanism helps to maintaining a balance between the loss and restoration of junctional adhesion^65^. Here we show that TEM of neutrophils through activated endothelial cells is significantly favoured by maintained EPLIN-α-controlled cell junction dynamics, which in turn is triggered by loss of junctional actin due to EPLIN-β-driven stress fiber formation.

## Results

### EPLIN recruits to actin docking structures induced by ICAM-1 clustering in endothelial cells, both in vivo and in vitro

**Fig. 1:**
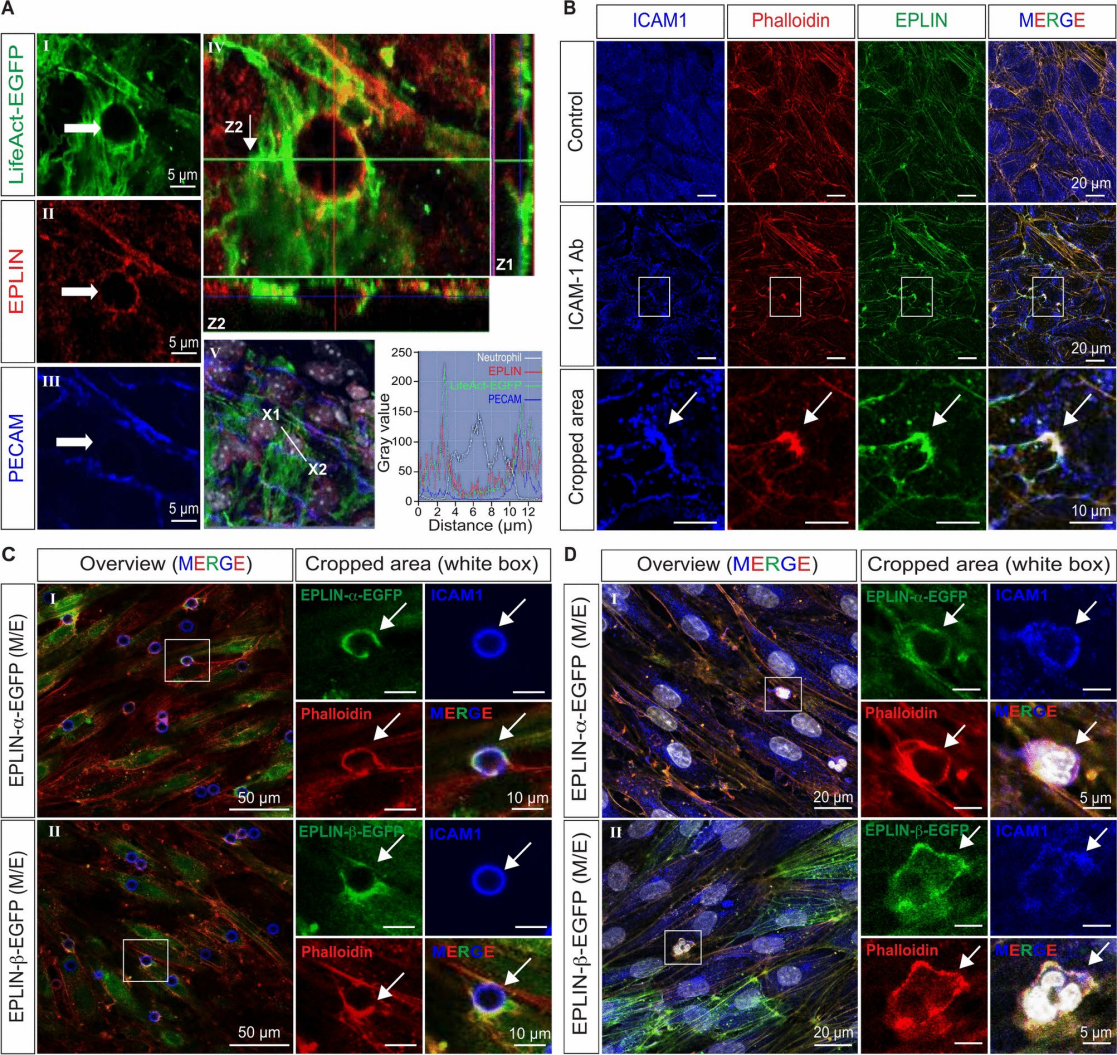
EPLIN is a component of actin filaments recruited to leukocyte transmigration sites and ICAM-1 clusters. (**A**) After irradiation of LifeAct-expressing transgenic mice followed by allogeneic transplantation of bone marrow, the mice were allowed to recover for 6 weeks. Transmigration of leukocytes was provoked in cremaster muscle by administration of 50 ng IL-1β for four hours. Mice were then euthanized, tissues was collected and laser scanning microscopy (LSM) was performed for the LifeAct-EGFP and immunolabelled EPLIN and PECAM and merged at higher magnification accompanied by Z-stacks as indicated (Z1-Z2). Lower image showes overview of LifeAct labelled inflamed cremaster muscle together with anti pan-EPLIN- and anti PECAM-1 antibodies, additionally superimposed with phase contrast images to identify the segmental nucleated neutrophils (white). Indicated line scan (X1-X2) verifies the colocalization of EPLIN and LifeAct-positive actin filaments at sites of leukocyte transmigration, whereas PECAM1 was irregularly labeled. (**B**) Confocal microscopy of antibody-induced ICAM-1 clusters in TNF-α-activated HUVEC. Detection of ICAM-1 (blue), EPLIN (green) by indirect immunostaining and actin filaments are labelled by phalloidin-TRITC (red) as indicated. Upper panel shows, control (no ICAM-1 Ab treatment). Middle panel shows ICAM-1 Ab treated cells. Lower panel displays the larger view of cropped areas (white boxes in the middle panel) of ICAM-1 clusters and co-labelled proteins as indicated. (**C**) Anti-ICAM-1-coated beads or (**D**) human neutrophils were added to TNF-α-activated HUVEC cultures expressing either EPLIN-α-EGFP or EPLIN-β-EGFP respectively (M/E = moderate expression). After fixation, cells were additionally labeled with phalloidin-TRITC (red) for actin filament and ICAM-1 antibody (blue) as indicated. In panel D, transmigrating neutrophils were identified by phase contrast microscopy due to their segmented polymorphic nuclei (white). The fluorescence intensity of the blue channel has been adjusted to improve the visibility of the corresponding label. The representative merged channel is presented as overview and the white boxes indicate the larger view of the cropped areas.

First, we tested whether EPLIN colocalises with actin structures of the endothelium during TEM in vivo. Male LifeAct-EGFP transgenic mice are irradiated with 920 rad, followed by bone marrow transplantation from wild type C57BL6 mice to differentiate between endothelial and neutrophil actin. After six weeks of recovery, mice are challenged by intra-scrotal injection of 50 ng IL-1β for four hours followed by tissue removal, fixation and immunolabelling. LifeAct-EGFP-positive endothelial docking structures colocalize with the anti-pan-EPLIN antibody, whereas the PECAM-1 antibody shows only irregular colocalization (Fig. 1A). Z-scans of the fluorescent labels at higher magnification confirm the colocalization between actin and EPLIN, as do line scans (Fig. 1A). These data suggest that EPLIN plays a role in leukocyte transmigration in vivo, most likely through its role in controlling actin dynamics. To elucidate the specific molecular mechanisms underlying the regulatory function of EPLIN-α and EPLIN-β in actin-mediated TEM, we start to use a TNF-α-activated human umbilical vein endothelial cell culture (HUVEC) model and evaluated neutrophil transmigration as the HUVEC model allows for genetic manipulation using siRNA approach and lentiviral-mediated gene transduction of fluorescence tagged EPLIN isoforms. Furthermore, the use of fluorescence-labelled EPLIN isoforms allows the analysis of the organisation and dynamics of different types of actin filaments in the endothelium. This is based on the observations that in endothelial cells EPLIN-α controls the dynamics of branched actin filaments at membrane protrusions through its interaction with the Arp2/3 complex, whereas EPLIN-β stabilises stress fibres but is not observed at membrane ruffles under physiological conditions^65^.

Similarly, as in vivo, anti-pan-EPLIN also co-localizes with actin filaments to anti-ICAM-1-induced ICAM-1 clusters in TNF-α-activated HUVEC cultures (Fig. 1B; Z-stack are shown in Supplementary Fig. S1). We further confirm in HUVEC cultures moderately expressing either EGFP-tagged EPLIN-α or EPLIN-β that both EPLIN-isoforms are components of actin filaments recruited to ICAM-1 clusters. Both fluorescently tagged EPLIN-isoforms, expressed at moderate levels, show the same actin pattern as in non-transduced cells, as previously shown in detail^65^. Fluorescence tagged EPLIN-isoforms localise to ICAM-1 clusters, regardless of whether the clustering is induced by ICAM-1-coated polystyrene beads (Fig. 1C; Z-stacks are shown in Supplementary Fig. S1), freshly isolated human neutrophils (Fig. 1D; Z-stacks are shown in Supplementary Fig. S1) or the promyelocytic leukemia cell line HL60 (Supplementary Fig. S1). These data show that actin filaments recruited by ICAM-1 cluster and during transmigration are positive for EPLIN-isoforms.

### Dynamic remodelling of actin-associated EPLIN isoforms in endothelial cells during neutrophil transmigration upon TNF-α activation

**Fig. 2:**
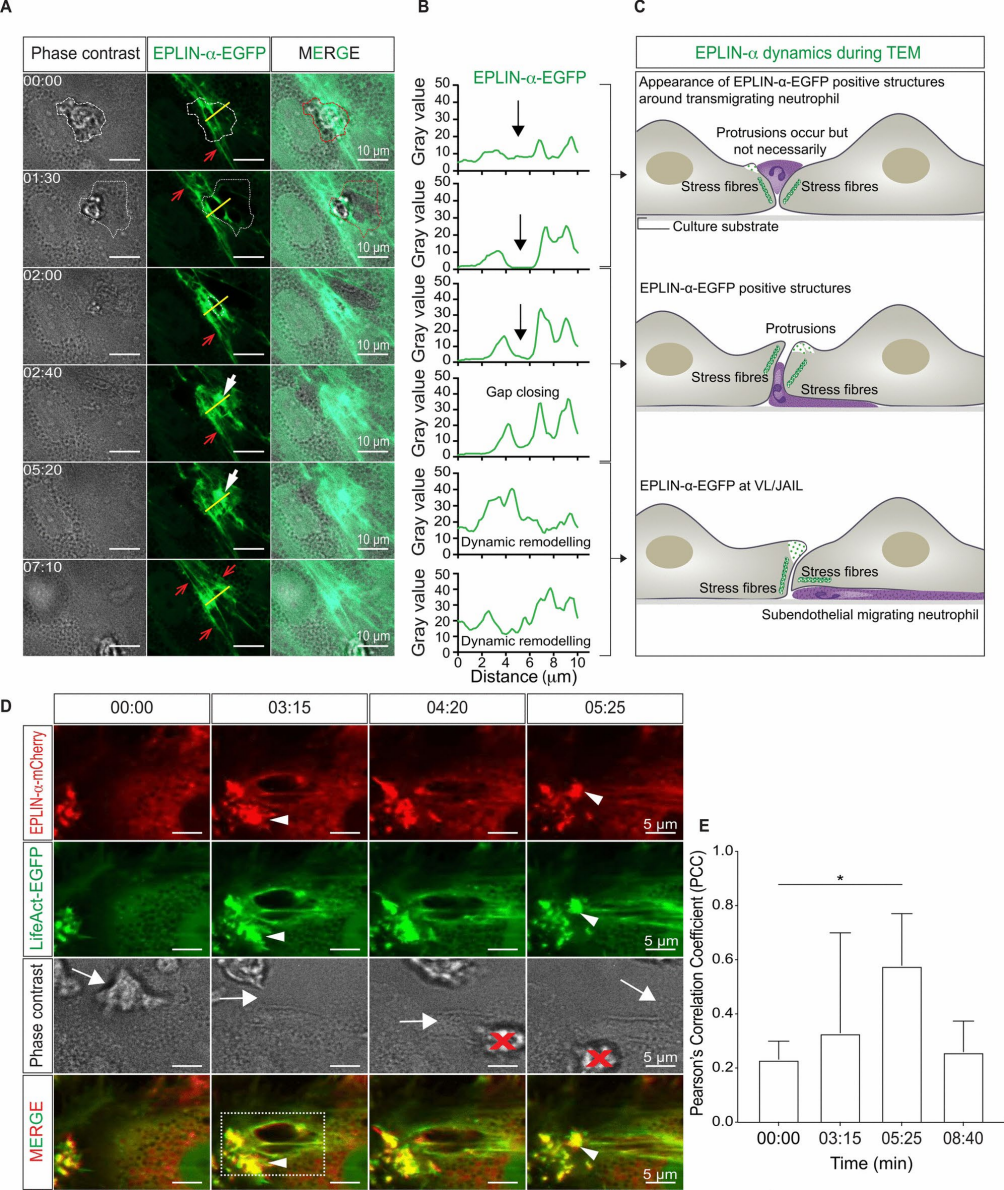
Dynamics of fluorescently labeled EPLIN-isoform in endothelial cells during neutrophil transmigration. (**A**) Fluorescence and phase contrast live cell images of transmigrating neutrophils through TNF-α-activated HUVEC monolayers moderately expressing EPLIN-α-EGFP. After leukocyte attachment (t = 00:00), EPLIN-α-EGFP is rapidly recruited to the gap where neutrophils adhere (dotted lines). Transmigration was nearly complete after t = 02:00. As expected EPLIN-α-EGFP also appears as a component of linear actin filaments (red arrows). Subsequently, extended EPLIN-α-EGFP structures appeared in the time range between 02:00–07:10, indicating membrane protrusions (white arrows) followed by gap closure (t = 02:00—07:10, mm:ss). (B) Illustrations of the dynamic process shown in A. (**B**) The indicated line scans show extended gap formation (black arrows) at time 01:30. Subsequently, from time 02:40 to 05:20, the intensity of EPLIN-α-EGFP increases, followed by upregulated dynamics leading to gap closure. (**C**) Illustrations of the dynamic process shown in A. EPLIN-α positive protrusions can occur at the onset of transmigration as part of docking structures, but consistently appear during pore closure. (**D**) Simultaneous expression of EPLIN-α-mCherry together with LifeAct-EGFP in HUVEC show colocalization of EPLIN-α and actin filaments during leukocyte transmigration. Arrow heads indicate colocalization of actin filament and EPLIN-α-mCherry at membrane protrusions (white arrowheads). The red cross indicates another leukocyte entering the image. White dashed area shows yellow spots indicating colocalization, but also show separations of EPLIN-α-mCherry and actin around the gap, characteristic for EPLIN-α dynamics as it moves behind branched actin filaments to terminate protrusions by binding to the Arp2/3 complex. (**E**) Pearson correlation coefficient analysis of three neutrophil transmigration sites shows transient increased colocalisation of EPLIN-α-mCherry and LifeAct-EGFP at 05:25, coinciding with pore closure followed by decrease. Unpaired t-test, *p* = 0.0409.

Activation of the endothelium by TNF-α is a proinflammatory stimulus as it induces ICAM-1 expression, which is critical for firm adhesion and subsequent TEM^30,66^. Furthermore, TNF-α also change endothelial morphology, accompanied by stress fiber formation and increased cell junction dynamics^14^. Branched actin filaments, controlled by EPLIN-α, drive junctional dynamics, whereas stress fiber assembly, primarily depend on EPLIN-β in endothelium^65^. Therefore, the role of EPLIN isoforms in TNF-α-induced actin dynamics and leukocyte TEM will be investigated using spinning disc live cell imaging. To this end, HUVEC cultures are transduced by lentiviral gene transfer to moderately express fluorescently tagged proteins, in particular EPLIN-α-EGFP, EPLIN-β-EGFP, LifeAct-EGFP and EGFP-p20 (ArpC4), a subunit of the Arp2/3 complex. Moderate expression of the respective fluorescence-labelled molecules has little or no effect on the physiological behaviour of actin organisation, whereas overexpression significantly modulates the actin dynamics of cells, as detailed in previous studies^65^. Activation of HUVEC cells by 50 ng TNF-α for 6 h does not alter the expression of EPLIN isoforms or the expression of the cell adhesion molecule VE-cadherin, as shown by Western blot analysis (Supplementary Fig. S2). To achieve high temporal and spatial resolution in the fluorescence live cell imaging approach, the studies are first performed under static conditions on EPLIN-α-expressing HUVEC to which neutrophils are added. Once the neutrophils are firmly attached, EPLIN-α-EGFP positive ring-like structures may form around the transmigration gap through which the neurophiles pass (Fig. 2A, Movie 1). Some of the EPLIN-α positive structures resemble transmigration cups as shown in Fig. 2D (Fig. 2A–D). Consistent with previous reports (for review see^67^), these cups are not observed in every case of neutrophil transmigration. After the neutrophils have completely crossed the endothelium, EPLIN-α-EGFP appear increasingly close to the transmigratory pores as curved structures, indicative for membrane protrusions (Fig. 2A–C; Movie 1), a phenomenon that was regularly observed. Line scans illustrate the dynamics of EPLIN-α (Fig. 2B) and the entire sequence of events are illustrated (Fig. 2C). In addition to the curved membrane protrusions, linear actin filaments resembling stress fibres also line the edge of the transmigratory pore (Fig. 2A–C). The presence of stress fibres has been suggested to limit permeability at this site^68^, and is consistent with EPLIN-β positive structure and dynamics (Movie 1). The association of EPLIN-α-mCherry with actin filaments during the process of transmigration is further investigated by co-expressing it with LifeAct-EGFP, a fluorescent marker for actin (Fig. 2D). Both tagged proteins colocalize at both the curved membrane protrusions near the pore and at the actin ring-like structures around transmigrating neutrophils. Pearson correlation coefficient analyses demonstrated a progressive colocalisation of EPLIN-α-mCherry and LifeAct over time together with increasing protrusions size for gap closure. Subsequently the signals decrease after gaps are closed (Fig. 2E). This assessment effectively captured the dynamic relationship between EPLIN-α-mCherry and LifeAct, highlighting their temporal association with protrusion formation and retraction during gap closure after TEM of neutrophils (Fig. 2D). These data strongly indicate that LifeAct-EGFP (actin) and EPLIN-α-mCherry are associated with the docking structure, if formed, as well as the membrane protrusions for pore closure.

**Fig. 3:**
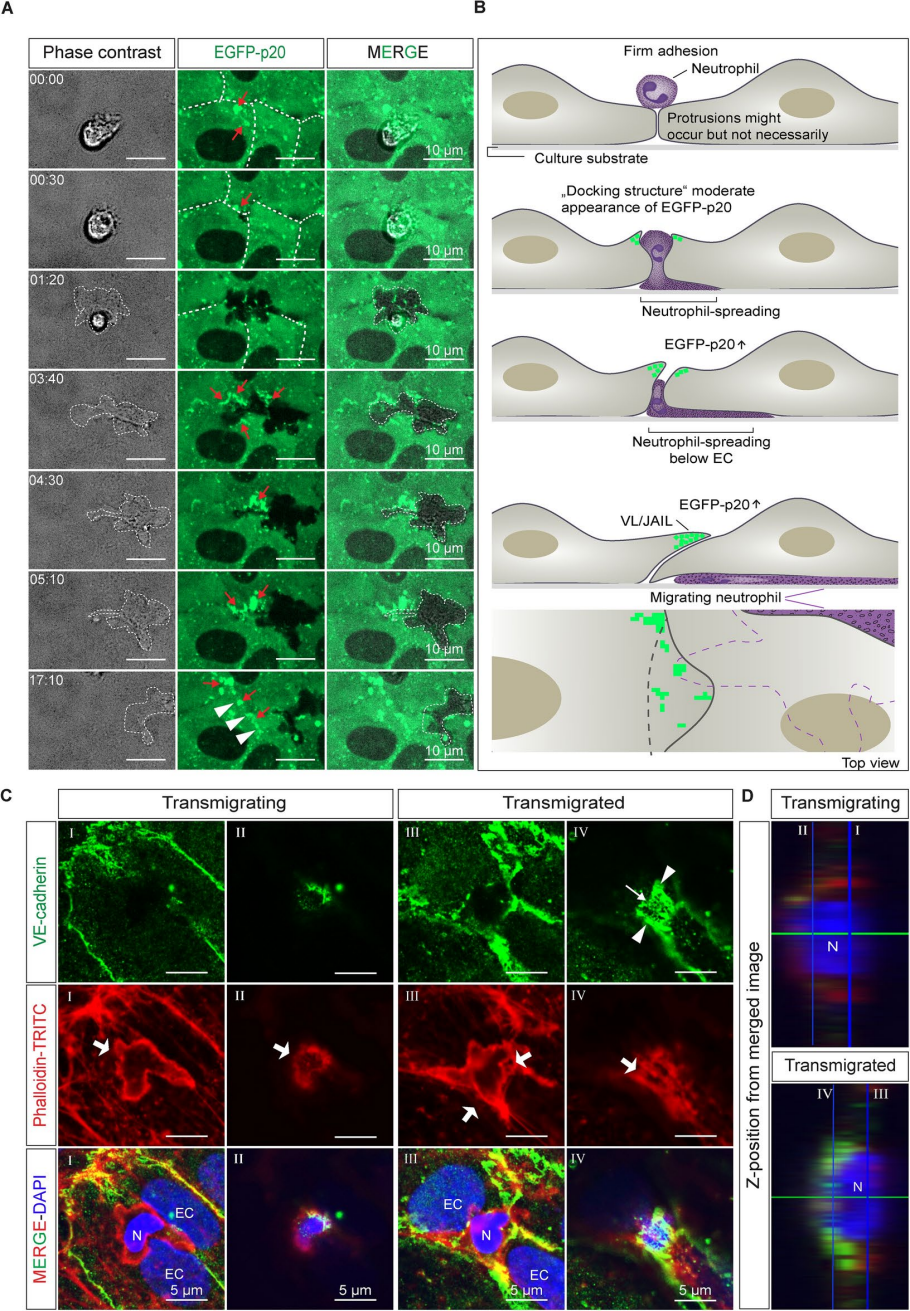
Time course of ARP2/3 complex dynamics during leukocyte transmigration analyzed by live-cell imaging. TNF-α-activated HUVEC monolayers moderately expressing EGFP-p20, a subunit of the ARP2/3 complex, were exposed to human neutrophils. (**A**) In confluent HUVEC, small EGFP-p20-positive protrusions (red arrows) appear transiently at the cell junctions (dashed white line at t = 00:00 & t = 00:30) and near leukocyte attachment sites (red arrows). During transmigration, neutrophils spread and moved below the endothelium (dotted lines). After the leukocyte had completely passed the endothelium, EGFP-p20 fluorescence (arrows) increased markedly (from t = 03:40), leading to closure of the leukocyte-induced gap. This process continued for several minutes (arrowheads), corresponding exactly to JAIL dynamics leading to “restitutio ad integrum” of the endothelial monolayer. The regular junctional dynamics of EGFP-p20, indicative of JAIL formation, is subsequently visible (t = 17:10). (**B**) The scheme illustrates the process observed by live cell imaging. (**C**,**D**) Immunolabeling of VE-cadherin and staining of actin filaments by phalloidin-TRITC before and after leukocyte transmigration in two z levels. During transmigration, VE-cadherin was largely absent from the pore, whereas after transmigration the pore was closed in a plaque-like manner (arrowhead) with VE-cadherin clusters (small arrow) consistent with branched actin induced JAIL dynamics (big arrows) restoring VE-cadherin adhesion. EC = nucleus of Endothelial Cell. N = nucleus of Neutrophils. Time code, mm:ss.

To further investigate whether branched actin filaments contribute to TEM, EGFP-p20, an EGFP-labelled subunit of the Arp2/3 complex, is expressed in HUVEC, since the ARP2/3 complex is essential for the formation of branched actin networks such as lamellipodia. Indeed, we find EGFP-p20 positive signals (indicative of the Arp2/3 complex) during leukocyte attachment in the early phase of transmigration, consistent with previously reported docking structures^22–24^. After neutrophils pass through the endothelial cells, EGFP-p20 structures appear near the transmigration pore, followed by pore closure (Fig. 3A, Movie 2). These data demonstrate that branched actin filaments controlled by the Arp2/3 complex drive membrane protrusions that are critical for pore closure. Consistently, we find negative VE-cadherin labelling at the transmigratory pore before TEM, whereas it is positive at this site after TEM (Fig. 3B and C). It should be noted that the activity of the Arp2/3 complex as well as the membrane protrusion activity increases throughout the layer after TNF-α treatment for 6 h which is related to increasing gap formation followed by gap closure. This suggests that pore closure is not an isolated event, but rather a widespread junctional phenomenon that also helps to maintain the basic integrity of the monolayer after TNF-α activation. Based on the data, we further propose that pore closure occurs via Arp2/3 complex-mediated VL/JAIL formation under the control of EPLIN-α to restore VE-cadherin-mediated cell adhesion after TEM.

**Fig. 4:**
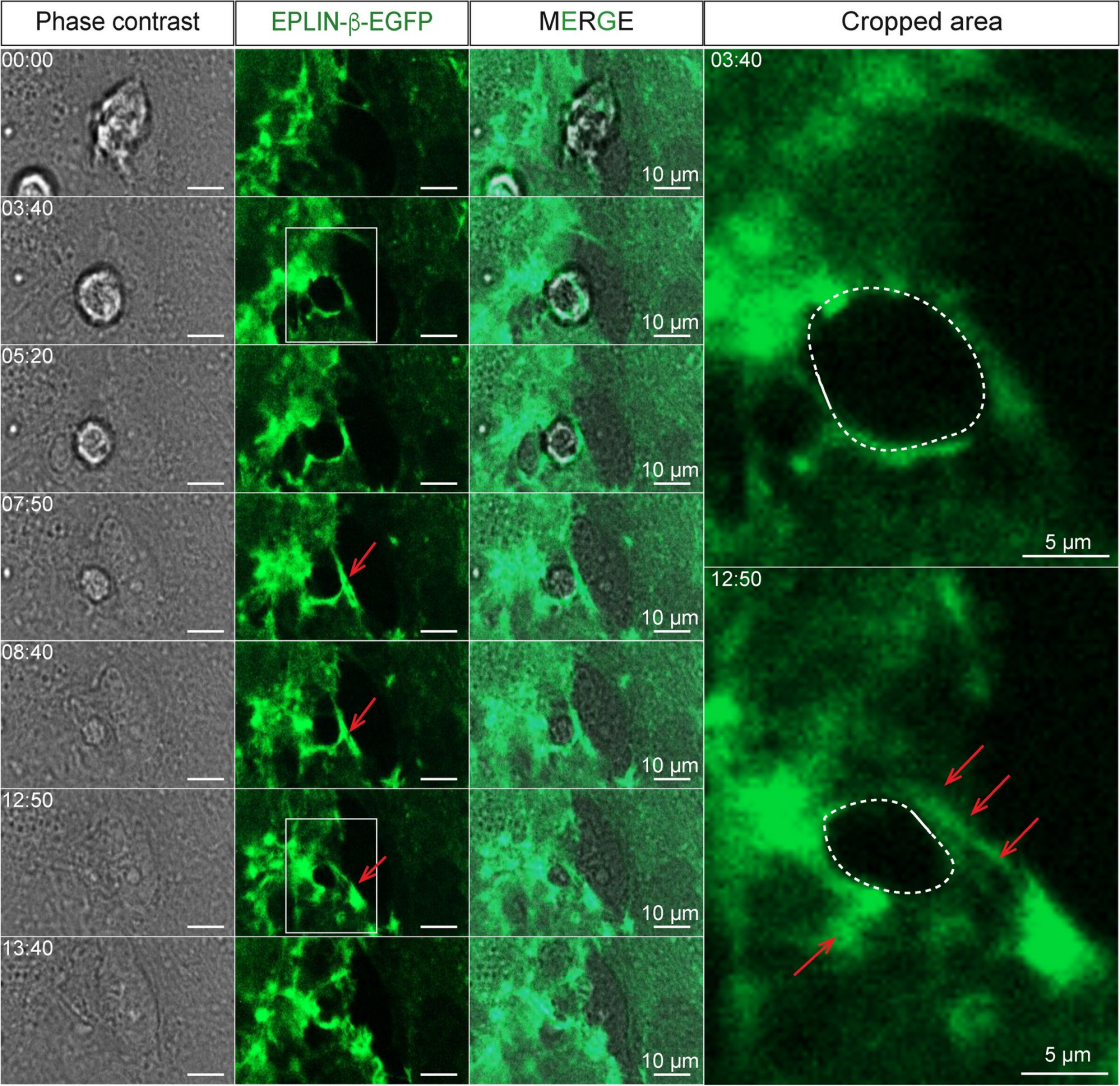
Fluorescence and phase-contrast live-cell imaging of transmigrating neutrophils through TNFα-stimulated HUVEC monolayers moderately expressing EPLIN-β-EGFP. After leukocyte attachment, EPLIN-β-EGFP was partially detected at the edge of leukocyte-induced gaps associated with linear actin filament stress fibres (red arrows), which were associated with narrowing gaps (dotted circles). Protrusion-like structures were not observed. Time code mm:ss.

EPLIN-β-EGFP, a stabiliser of contractile actin bundles^64,65,69^ such as stress fibres, is also found near the transmigration pore in TNF-α activated HUVEC, which forms rapidly after neutrophil attachment and persists during TEM (Fig. 4). The persistence of stress fibres during TEM is also consistent with a much lower turnover of EPLIN-β in actin bundles compared to EPLIN-α, as shown by FRAP analysis (see below and^65^) as well as with the proposed sealing function of stress fibres at the transmigratory pore^18,70^. Together, the data strongly indicate a critical role of EPLIN-β in TNF-α-mediated stress fiber formation and weakening of cell junctions, which in turn increases junction dynamics maintained by EPLIN-α.

### EPLIN-mediated, specific control of actin dynamics is required for neutrophile transmigration under shear stress

**Fig. 5:**
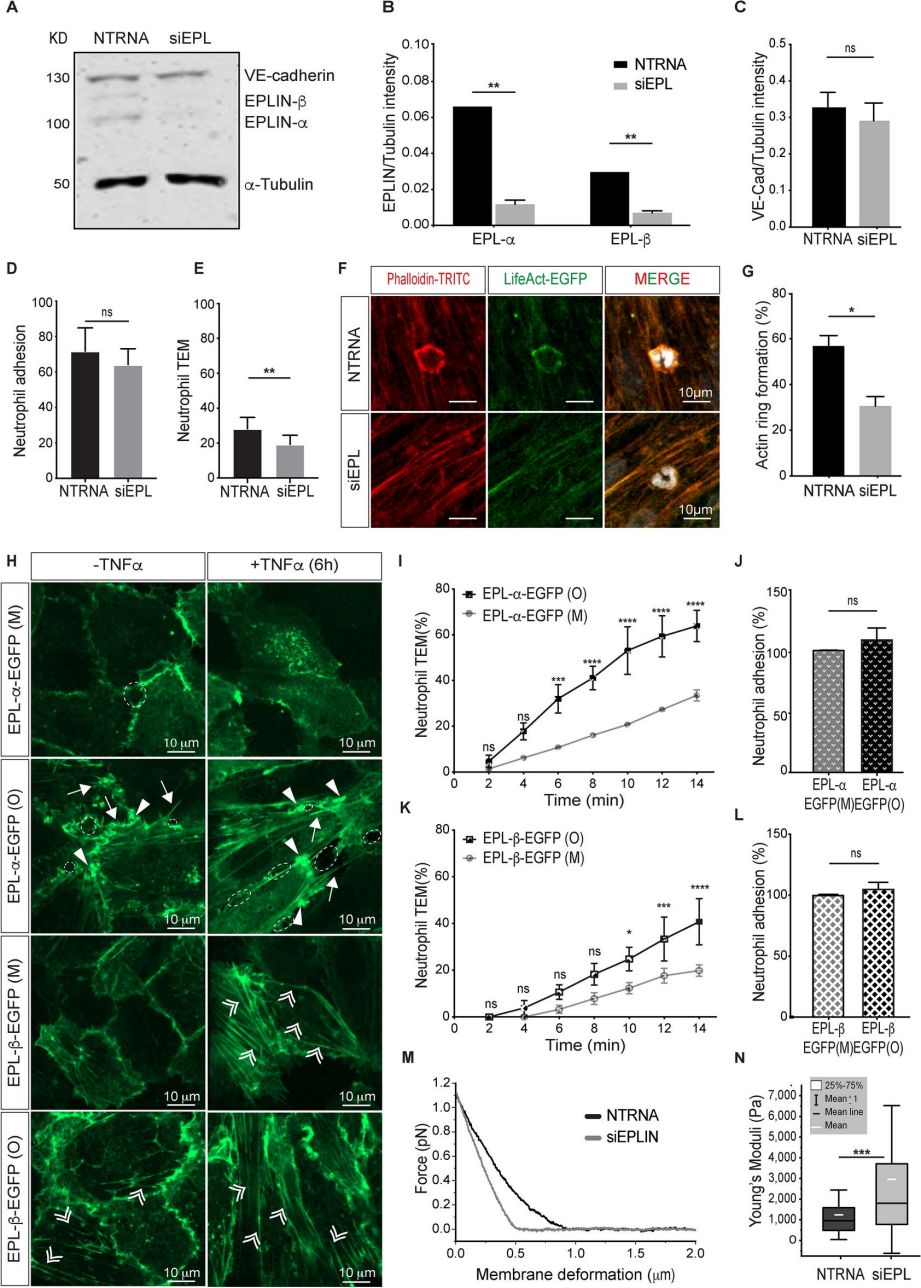
EPLIN isoforms modulate leukocyte transmigration. (**A** to **C**) Western blot analyses as indicated (n = 3). α-Tubulin, internal control. Significance was tested using unpaired Student’s t-test. (Full immunoblot is shown in supplementary Fig. S6, the brightness of original blot is adjusted using Fiji). (**D**) Neutrophil adhesion and (**E**) transmigration in EPLIN-depleted (siEPL) and control HUVEC (NTRNA) after TNF-α (50 ng/ml) under flow. Adherens of neutrophils in NTRNA treated HUVEC (71.8 ± 4,157) was not significantly different from siEPL-treated cells (64.2 ± 2,812; *p* = 0,1473 unpaired t-test). TEM of neutrophils in control HUVEC (28.2 ± 2,054) and siEPL- treated HUVEC (19.3 ± 1,654; *p* = 0.0034). Unpaired t-test following Shapiro–Wilk normality test. A total of 1,404 neutrophils in NTRNA cells and 844 neutrophils in siEPL cells were counted after 6 min from 4 independent experiments. (**F**) LifeAct-EGFP expressing HUVEC were treated with NTRNA or siEPL respectively. After application of neutrophils cultures were stained with phalloidin-TRITC (red) and DAPI labels the segmented neutrophiles (white). (**G**) Actin ring-like structure formation in NTRNA- or siEPL-treated HUVEC. 100 cells were analyzed from 3 independent experiments. Error bars ± SEM. *, *p* = 0,05; **, *p* = 0,005. (**H**) HUVEC expressing tagged-EGFP EPLIN isoforms upon TNF-α stimulation. Arrows indicate spikes due to abortive JAIL. Arrow heads indicate membrane protrusions. Double arrow heads indicate stress fibres. (**I**–**L**) TEM of adherent and transmigrating neutrophils respectively at different time points under flow (1dyn/cm^2^). (M) moderate expression, (O) overexpression, upon treatments as indicated. One-way ANOVA test was done for the statistical analysis of multiple groups. *P* < 0.0001) 150–200 Neutrophils from 3 independent experiments were analyzed. (**M**) Force–displacement curves (approximation) of NTRNA- and siEPL- treated HUVEC. Note the steeper slope in the siEPL- (gray line) compared to NTRNA-treated cells (black line). (**N**) Box plot of the elastic moduli (Young´s moduli) from AFM measurements in NTRNA- (black) and siEPL-treated (grey) HUVEC. Mann–Whitney test revealed a statistically significant difference (*p* < 0.0005) as indicated. Black horizontal line: median; white lines: mean; whiskers: STDEV; The following values (in Pa) were identified for siEPL (n = 1428): Q1 = 772, Q3 = 3710, mean ± SEM = 2951 ± 3569 and for NTRNA (n = 1391): Q1 = 449, Q3 = 1554, mean ± SEM = 207 ± 1200. Median = siEPL 1801, Median NTRNA = 924.

Using siRNA-mediated EPLIN knockdown in HUVEC, the role of EPLIN in neutrophil transmigration under physiological shear stress conditions is investigated. Specifically, HUVEC are treated with anti EPLIN siRNA (siEPL), which reduces the expression of EPLIN-α by 85% and that of EPLIN-β by 75% (Fig. 5A and B). The expression of VE-cadherin is not affected by treatment with either siEPL or non-target RNA (NTRNA) (Fig. 5C). In NTRNA treated confluent HUVEC (1–1,2 × 10^5^ cells/cm^2^) junctional actin together with VE-cadherin are largely restricted to cell junctions while application of TNF-α leads to stress fiber formation and a loss of junctional actin (Supplementary Fig. S2). In contrast, cells treated with siEPL show irregular actin filaments, some of which appear at the cell periphery (Supplementary Fig. S2). Application of TNF-α shows shape change, but largely fails to produce characteristic cytoplasmic stress fibres (Supplementary Fig. S2). In addition, there is an apparent increase in JAIL size following siRNA treatment. The data confirm the inhibitory role of EPLIN-α in terminating branched actin filament extension via Arp2/3 complex interaction and support the role of EPLIN-β in promoting stress fibre formation, as previously analysed in detail^65^.

These EPLIN-depleted HUVEC are next investigated for their ability to allow TEM of neutrophils under unidirectional laminar flow of 1 dyn/cm^2^ using a novel, self-constructed “insert flow chamber” (patent No 10 2020 131 894). The chamber is designed to culture the cells in regular standard conditions completely avoiding nutrition or oxygen depletion. The chamber provides a unidirectional laminar flow, as proved by both numerical simulation (Supplementary Fig. S3) and dye flow experimentation respectively (personal communication Hans Schnittler)^71^.

Application of freshly-isolated human neutrophils to the chamber results in neutrophil adherence to both siEPL-treated and NTRNA-treated HUVEC (Fig. 5D). In contrast, TEM of neutrophils is reduced about 1.5-fold in siEPL-treated HUVEC compared to NTRNA-treated cells (Fig. 5E). In addition, laser scanning microscopy (LSM) shows that 57% of migrating neutrophils are associated with a complete actin ring, whereas only 30% are found in siEPL-depleted HUVEC (Fig. 5F, G). These data indicate that EPLIN isoforms play an important role in leukocyte transmigration by controlling actin ring formation.

To further differentiate the role of EPLIN isoforms in leukocyte TEM, EPLIN-α-EGFP and EPLIN-β-EGFP are selectively overexpressed or moderately expressed, respectively, in HUVEC by viral titration (Supplementary Fig. S3). Despite viral transduction, the TNF-α-mediated expression level of ICAM-1 remains unchanged (Supplementary Fig. S3). Interestingly, the TNF-α-induced cell shape change occurs under all conditions (Fig. 5H). Fluorescence-labelled EPLIN-α and EPLIN-β preferentially label curved membrane protrusions and linearly arranged stress fibres, respectively, allowing differentiation between these actin types in the endothelium. Moderate expression of EPLIN isoforms is consistent with an actin pattern identical to that of untransduced cells, whereas overexpression has pronounced effects on actin organization and dynamics, as previously characterized in detail^65^. While moderate EPLIN-α expression decorates membrane protrusions, EPLIN-α overexpression leads to an early abortion of curved membrane protrusions (most likely JAIL) due to its inhibitory function at this site, while filopodia formation increases, accompanied by intercellular gap formation together with a moderate development of stress fibres^65^. These effects also occur after TNF-α application and examples of these effects on actin organisation are shown in Fig. 5H. The changes in actin organization and dynamics induced by EPLIN-α overexpression are accompanied by a significant increase in TEM of neutrophils under shear stress of 1 dyn/cm^2^ (Fig. 5I), while neutrophil adhesion remains largely unchanged (Fig. 5J). Therefore, it can be proposed that altered actin mediated junctional dynamics associated with decrease in barrier function^65^ provide additional exit points for neutrophils. Moderate expression of EPLIN-β in confluent HUVEC shows limited amounts of EPLIN-β-positive stress fibres (Fig. 5H), whereas overexpression of EPLIN-β shows prominent stress fibres after TNF-α treatment (Fig. 5H), consistent with previous reports^65^. This is in contrast to the overexpression of EPLIN-α and reflects the stress fiber stabilizing role of EPLIN-β, while junctional dynamics remain largely intact^65^. Under these conditions, overexpression of EPLIN-β only moderately increases leukocyte TEM (Fig. 5K), while leukocyte adhesion remains unchanged as well (Fig. 5L). Importantly, the TEM rate through HUVEC overexpressing EPLIN-α is approximately 68%, whereas the TEM through HUVEC overexpressing EPLIN-β cells reaches approximately 40%. The lower TEM rate of neutrophils observed in EPLIN-β-EGFP overexpressing endothelium compared to EPLIN-α-EGFP overexpression can be explained by the stabilising effect of stress fibres, which consume G-actin and thus shift the G- and F-actin ratio^72^. As a result, junctional actin is reduced, which still allows for the dynamics of autoregulated and EPLIN-α controlled protrusion formation and thus dynamic maintenance of barrier function^65^. These data highlight the importance of EPLIN isoforms in the spatial and temporal control of actin dynamics during neutrophil TEM.

### EPLIN-mediated alteration of actin dynamics alters biophysical properties in HUVEC

Since actin dynamics alter the biophysical properties of the endothelium^11,73^, and the biophysical properties of the endothelium are also modulated during TEM^33,74^, we used atomic force microscopy (AFM) to search for EPLIN-dependent changes in endothelial elasticity (Young’s modulus). Measurements were taken at the highest point of the cell (core/perinuclear region) to ensure consistent mechanical characterisation of the EPLIN influence, as cell stiffness is critical for TEM^33^. Young´s modulus is multifactorially determined e.g. by the cytoskeleton, including actin/myosin-mediated contraction and cytoplasmic viscosity^75–79^. AFM force–displacement measurements show a significant difference in the indentation curves between EPLIN-depleted and control endothelial cells (Fig. 5M). Furthermore, siEPL-treated cells (median = 1802 Pa) compared to NTRNA-treated controls (median = 924 Pa) display a significant increase in Young´s modulus as determined by the Hertz model^80^, (Fig. 5N). It should be noted that a high Young’s modulus corresponds to high elasticity and therefore low softness. The increased endothelial stiffness after EPLIN depletion most likely alters overall actin dynamics, including junctional dynamics, and contributes to the downregulation of TEM. The data suggest that EPLIN-driven maintenance of actin dynamics is critical for endothelial stiffness and hence leukocyte transmigration. This is consistent with the observation that leukocyte transmigration occurs at sites associated with low endothelial stiffness^33^.

### Re-expression of EPLIN isoforms in CRISPR/Cas9-mediated EPLIN knockout EA.hy cells confirm the specific roles of different actin filament types in TEM

CRISPR/Cas9 technology is used to knock out the EPLIN-α and EPLIN-β genes in EA.hy 926 endothelial cell lines, to precisely characterise the role of EPLIN-α and EPLIN-β in TEM. WT-EA.hy 926 endothelial cell line are derived from fusing primary HUVEC with the epithelial cell line A549. EA.hy 926 cells (further referred as EA.hy) share important characteristics with native endothelial cells and are employed for endothelial cell biology studies^81^, particularly they show responses similar to HUVEC when stimulated with TNF-α^82^. EPLIN is expressed as two isoforms from a single LIM domain and actin binding (LIMA) gene located at chromosome 12q13 under the control of alternative promoters^63^. The gene is disrupted simultaneously by targeting either exon 4 or exon 8, and subsequently obtained cell clones are screened for the absence of EPLIN expression by Western blotting using an anti-EPLIN antibody (Supplementary Fig. S4). Next, the lack of respective wild-type alleles from selected clones is confirmed by TIDE analysis of Eplin-KO-derived DNA sequences (Supplementary Fig. S4). Finally, two of the clones (KO #6 and KO #26) are applied for further characterization and subsequent analyses of leukocyte TEM. The expression levels of selected proteins associated with the cytoskeleton, cell junctions and substrate adhesions are analyzed in CRISPR/Cas9-mediated Eplin-KO EA.hys by Western blotting. The data show that the levels of VE-cadherin, α-, β- and γ-Catenin , Vimentin, Vinculin and Paxillin are comparable in Eplin-KO and WT EA.hys (Supplementary Fig. S5).

**Fig. 6:**
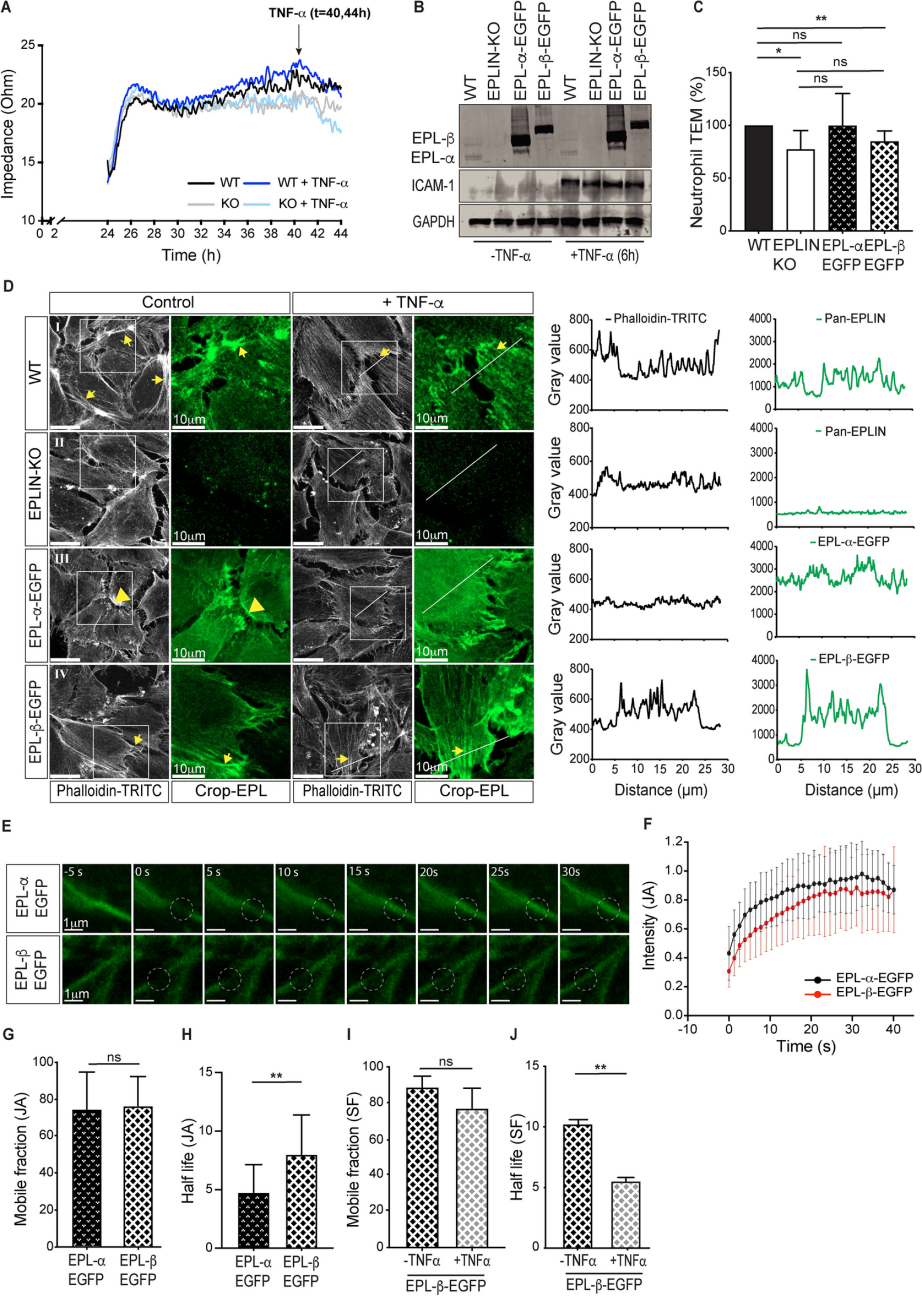
Characterisation of EA.hy cell lines and the influence of the EPLIN isoforms on transendothelial migration of neutrophils. (**A**) Transendothelial resistance of EA.hy WT and KO cells during cell growth. (**B**) Western blot analysis of EA.hy cell lines before or after re-expression of each of the EPLIN isoforms and subsequent activation by 50 ng/ml TNF-α. GAPDH served as an internal control. Full immunoblots are shown in supplementary Fig. S6 (**C**) The indicated EA.hy cell lines were cultured on trans-well filter membranes. TEM of neutrophils was determined by counting of transmigrated neurophils from the lower compartment. TEM is reduced in Eplin KO cells (*p* = 0.0425), while re-expression of EPLIN-α-EGFP (EPL-α-EGFP cells) returned TEM to control levels, seen in WT EA.hy cells (*p* = 0.7893). In contrast the re-expression of EPLIN-β-EGFP (EPL-β-EGFP) showed only a moderate increase in TEM but still remained significantly lower compared to WT EA.hy cells (*p* = 0.0306). Data were analyzed from 4 independent experiments. Error bars represent ± SEM, *, *P* < 0,05. **, *P* < 0,005. ns = not significant. Kruskal–Wallis test was done for the multi comparison of different groups. (**D**) EA.hy cell lines as indicated were analysed by LSM. Actin filaments were labelled with phalloidin-TRITC (white). EA.hy WT and EA.hy KO cells did not express fluorescently-labelled proteins and were therefore immunolabelled indirectly using anti-pan-EPLIN (green channel). Re-expression of EPLIN-α-EGFP and EPLIN-β-EGFP in EA.hy cells as indicated (cropped accordingly). EPLIN labelling has also been cropped as indicated. Actin filaments (in black) and EPLIN labelling (in green) are shown at the corresponding positions in the line scans. Images shown are representative of three independent experiments. Scale bar, 10 µm. (**E**–**J**) FRAP analysis of EPL-α-EGFP, F and EPL-β-EGFP locolized near junctional actin filaments (JA) in the EA.hy KO cells. (**E**) Representative images of a FRAP experiment in EA.hy KO cells expressing EPL-α-EGFP (upper panel) or EPL-β-EGFP (lower panel). Scale bar, 1 μm. (**F**) Mean values of the normalized fluorescence recovery intensities ± SEM of EPL-α-EGFP (n = 27 areas) and EPL-β-EGFP (n = 31 areas), from 4 independent experiments. (**G** and **H**) Quantification of mobile fraction and half life of the EPL-α-EGFP or EPL-β-EGFP (*p* = 0.0020). (**I** and **J**) Quantification of mobile fraction and half life of the EPL-β-EGFP localized at SFs, upon TNFα stimulation. (unpaired t-test, *p* = 0.0087).

The functional ability of the WT and KO EA.hy cell line (#clone 6) to form an integral monolayer and their response to TNF-α stimulation, both being critical parameters for TEM, were investigated by determining the transcellular electrical resistance through impedance spectroscopy measurements^83^. For this, the cells are seeded at a density of 5 × 10^4^ cells per well on filters with a pore size of 3 µm and are allowed to settle for 24 h. We find a progressive increase in resistance in both cell lines, peaking between 25 and 27 h after seeding (Fig. 6A). However, monolayer formation and integrity differ between the two cell types. The resistance of KO monolayers reaches a persistent plateau after about 28 h, whereas the TER of the WT cells continues to increase significantly (Fig. 6A). In addition, the administration of TNF-α leads to a significant reduction in the electrical resistance in both cell lines (Fig. 6A). The data demonstrate that EPLIN-isoforms are required to fully establish a barrier-forming monolayer. However, the TNF-α lowering effect on TER also appears in EPLIN-KO and WT cells, while ICAM-1 expression is still observed. (Fig. 6B), showing an appropriate TNF-α response in EA.hy cell lines.

### EPLIN-isoforms specifically modulate neutrophile transmigration in EA.hy cell lines

TEM of neutrophils is evaluated using confluent WT EA.hy cells, Eplin KO-EA.hy as well as Eplin KO cells re-expressing either EPLIN-α-EGFP (EPL-α-EGFP) or EPLIN-β-EGFP (EPL-β-EGFP) in transwell filter assay. Re-expression of EPLIN-isoforms leads to significant expression levels (Fig. 6B). The filter assay was previously successfully used for studying TEM through EA.hy cells^84^. Filter assays allow for easier determination of leukocyte TEM, as EA.hy cells have a more heterogeneous morphology compared to HUVEC, making counting based on image analysis difficult. TEM of neutrophils shows a decrease of approximately 22.5% in Eplin KO EA.hy cells compared to WT EA.hy cells, which is consistent with siRNA mediated EPLIN depletion in HUVEC. Restoration of EPLIN-β expression in these cells had minimal to no effect on TEM of neutrophiles. Furthermore, while HUVEC overexpressing EPLIN-α showed a trend towards increased transmigration compared to knockout (KO) cells, this difference did not reach statistical significance (Fig. 6C). This may be due to the inherent variability of the EA.hy model, the absence of EPLIN-β affecting actin dynamics, and the potential imbalance of EPLIN-α at cell junctions in rescue cells. These factors suggest a complex interplay between EPLIN isoforms that requires further investigation beyond the scope of this study.

In order to find morphological correlates of these functional data, the actin pattern of the different EA.hy cell lines is compared using laser scanning microscopy. WT cells exhibited prominent stress fibres and characteristic actin membrane protrusions. The EA.hy KO cells lacks the prominent actin cables but still displayed cytoplasmic thin actin fibres, some of which were aligned parallel to the cell borders (Fig. 6D). The actin filaments largely colocalized with anti pan-EPLIN antibodies in WT cells, whereas KO cells show negative staining. Re-expression of EPLIN-α-EGFP resulted in only a subtle change in the actin pattern compared to KO cells, with actin filaments at cell contacts being more pronounced which is likely to be related to JAIL. Conversely, re-expression of EPLIN-β-EGFP resulted in stress fiber formation, although less prominent than in WT cells (Fig. 6D). However, a significant change in the actin pattern in this cell line was observed upon treatment with TNF-α. While a significant increase in stress fibres is observed in WT cells, KO cells also show small stress fiber induction but lack thick actin bundles (Fig. 6D). Similarly, TNF-α stimulation in EPLIN-α re-expressing cells only resulted in an accumulation of actin and EPLIN-α-EGFP at cell contacts with filopodia-like protrusions (Fig. 6D). In contrast, re-expression of EPLIN-β-EGFP resulted in more evident stress fiber formation after TNF-α treatment (Fig. 6D). These results are consistent with modulations of EPLIN-isoforms in HUVEC.

### EPLIN-isoforms modulate actin dynamics but not contractility

To further explore EPLIN-isoform-dependent actin dynamics, EA.hy KO cells re-expressing either EPLIN-α or EPLIN-β tagged to EGFP (EPL-α-EGFP, EPL-β-EGFP) were subjected to fluorescence recovery after photobelaching (FRAP). In particular, FRAP analyses is performed on junctional actin under control conditions in EA.hy after re-expression of EGFP-tagged EPLIN-isoforms (Fig. 6E and F). No differences were observed in the mobile fractions of EPLIN-α-EGFP and EPLIN-β-EGFP (Fig. 6G). In contrast EPLIN-β-EGFP shows a significant higher half-life time (prolonged recovery time) (Fig. 6H), which is consistent with a stabilizing role of EPLIN-β on actin bundles, including the junctional actin, while EPLIN-α controls actin remodelling at cell junctions. FRAP analysis was also performed upon TNF-α stimulation on stress fibres in EPLIN-β-EGFP re-expressing cells. In this investigation EPLIN-α-EGFP cells were excluded as insufficient stress fiber development occurs during TNF-α stimulation, precluding a direct comparison. TNF stimulation did not affect the mobile fractions (Fig. 6I). However, it reduced the half-life of EPLIN-EGFP molecules on stress fibres (Fig. 6J), consistent with TNF-induced stress fibre formation. These data, obtained using EA.hy cell lines re-expressing either EPLIN-α-EGFP or EPLIN-β-EGFP, highlight the specific role of EPLIN-β in stabilising actin bundles, whereas EPLIN-α regulates actin dynamics at junctions.

In addition, a previous report indicates that stress fiber contraction plays a role in barrier function control during TEM of neutrophils^18,70^. Thus, stress fiber contractility was measured by laser ablation experiments in the EA.hy cell lines. Stress fibres containing fluorescently-labelled EPLIN isoforms were cleaved and subsequently, time-lapse recordings were taken at a 200 ms interval. Image sequences were then used to measure the retraction velocities and subsequently determination of the stress fibres contractility using the Kelvin body model (Kumar et al., 2006). Notably, stress fiber tension in the distinct EA.hy cell lines was independent of the expression of the EPLIN isoforms (Supplementary Fig. S5). These data sugest that actin dynamics rather than contractility of stress fibres is differentially modulated by EPLIN-isoforms.

## Discussion

**Fig. 7:**
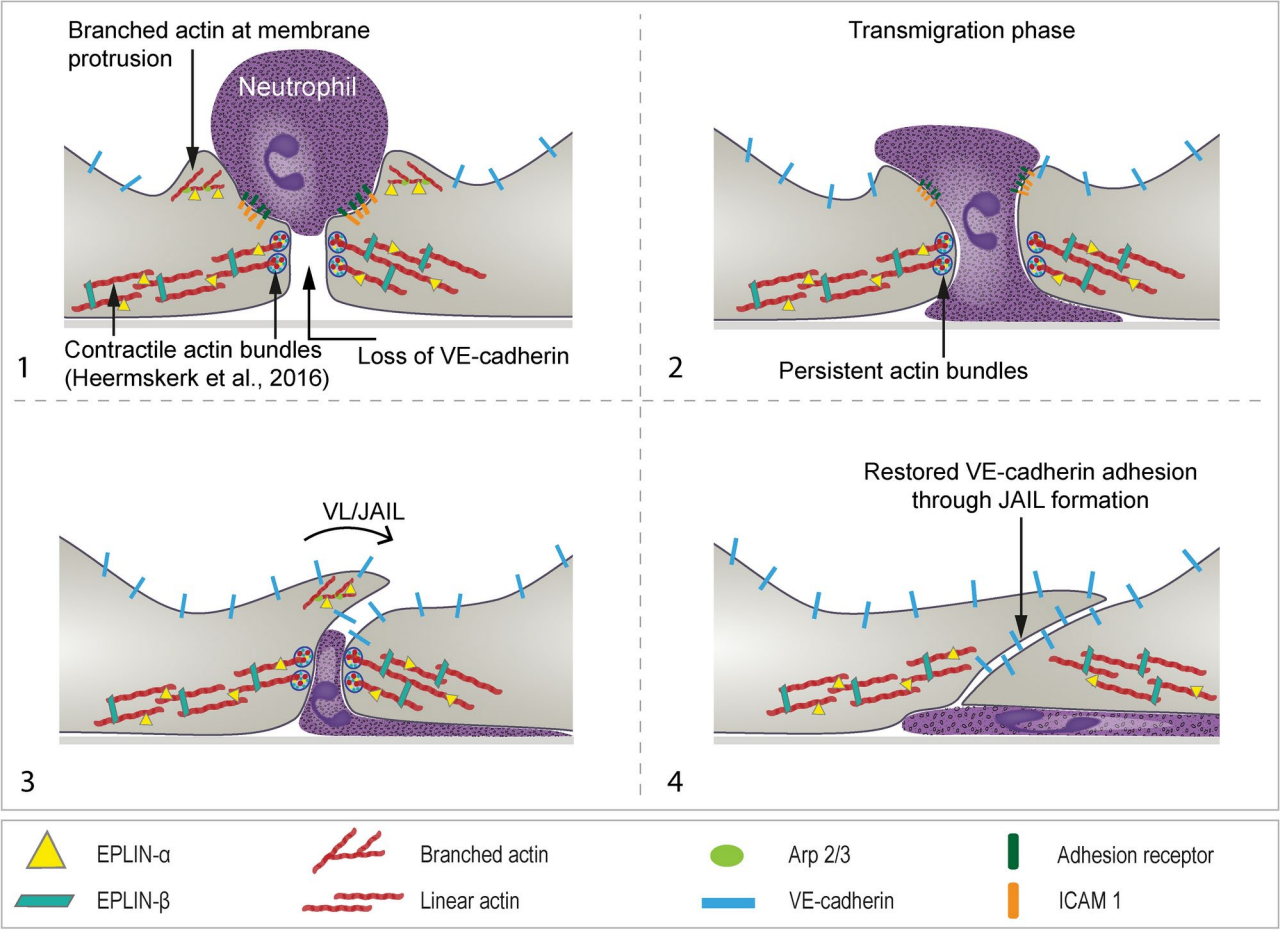
Scheme, illustrating the proposed series of events during leukocyte transmigration through activated endothelium in an EPLIN-dependent manner. (1) After the neutrophils firmly adhere to the activated endothelium, branched actin filaments develop under the control of EPLIN-α, forming docking structures. At the same time, the neutrophils begin to penetrate through a gap that is free of VE- cadherin. (2) During transmigration of the leukocyte EPLIN-β stabilizes actin bundles sourrounding the gaps (blue circles) to most likely seal the gaps protecting the endothelium from uncontrolled leakage, and persist until leukocyte transmigration is completed. (3) It appears that VL/JAIL are already formed in the terminal phase of leukocyte transmigration, triggered by the Arp2/3 complex and controlled by EPLIN-α. (4) After leukocyte transmigration is complete, VL/JAIL form with increasing frequency, a process that ultimately closes the transmigration gap.

Actin dynamics are essential for the efficient and coordinated movement of leukocytes across the endothelial barrier to reach sites of inflammation^24,68,85^. Here we focused on specific types of endothelial actin filaments, branched actin-driven membrane protrusions and actin bundles like stress fibres which are controlled by EPLIN-isoforms^62,64,65,86^ during transendothelial migration (TEM) of neutrophils. TNF-α induces endothelial cell adhesion molecules^5,19,87,88^, alters cell shape and enhances actin dynamics^14,35,36,89,90^. Fluorescently labelled EPLIN isoforms distinguish actin filament types in HUVEC. In particular, EPLIN-α labels curved, branched networks, whereas EPLIN-β labels straight actin bundles like stress fibres. By manipulating EPLIN-α and EPLIN-β^64,91^, regulators of endothelial branched actin filaments and stress fibres respectively^62,65^, we demonstrated their specific, spatially restricted control of actin dynamics during neutrophile TEM, as illustrated (Fig. 7). This includes the effect of EPLIN in determining increased stiffness following EPLIN-isoform depletion, consistent with previous findings^33^. Although accurate measurement of endothelial cell junctions was not possible due to their flat morphology and the lateral dimensions of the indenter, the overall effect of EPLIN depletion on endothelial stiffness is evident.

### Our work provides three new main insights into the process of neutrophil transendothelial migration

#### First, EPLIN isoforms are important for TNF-α-mediated formation of stress fibres, increased permeability and maintenance of cell junction dynamics

EPLIN-β is essential for TNF-mediated stress fibre formation as demonstrated in EPLIN siRNA-treated HUVEC and CRISPR-Cas EPLIN KO EA.hy cells. Stress fibre formation weakens endothelial barrier function, creating small gaps required for neutrophil passage^92–96^ and facilitating leukocyte transendothelial migration (TEM). This is consistent with tightening of junctions that inhibits TEM^97^.

EPLIN-β-mediated stress fibre induction by TNF-α increases junctional dynamics, whereas EPLIN-α maintains these dynamics by terminating junction-associated intermittent lamellipodia (JAIL) through inhibition of the Arp2/3 complex^65^. This is consistent with the Rac/WAVE/WASP activated Arp2/3 complex, regulating endothelial barrier function and leukocyte transmigration^39,98–100^. Recent research suggests that Arp2/3 may also contribute to the regulation of linear actin filaments when activated by SPIN90/Dip family proteins^45^, the role of which in TEM of neutrophils is unclear. While protrusions are common at transmigratory pores, linear actin filaments such as stress fibers with EPLIN-β and EPLIN-α may also assist neutrophil transendothelial migration by facilitating docking structure formation, gap sealing and pore closure. The return of VE-cadherin to transmigratory gaps after TEM suggests JAIL-mediated gap closure, as JAIL are defined by its direct ability to restore VE-cadherin adhesion^60^. EPLIN-β controls stress fibre formation, affecting cell contact dynamics and barrier function after TNF-α treatemnet, whereas EPLIN-α fine-tunes cell junction dynamics, balancing contact loss and recovery during neutrophil TEM. Furthermore, EPLIN isoforms cooperatively regulate actin remodelling after TNF-α treatment, as suggested by their presence at transmigration sites in vivo and demonstrated in HUVEC cell cultures. However, the effects of EPLIN isoforms on transmigration of other types of leukocytes need to be assessed and their effects in vivo require further investigation.

#### Second, EPLIN-α and EPLIN-β work together to regulate endothelial cell dynamics through their different interactions with actin structures

EPLIN-regulated branched actin filaments and actin bundles play distinct roles in leukocyte transmigration. Experiments involving siRNA-mediated depletion, overexpression and selective re-expression of EPLIN isoforms in different cell types revealed that EPLIN-α-mediated control of junctional dynamics is crucial for neutrophils to find transmigration exit sites. Overexpression of EPLIN-α in HUVEC doubled the rate of transendothelial migration due to altered junctional dynamics and increased intercellular gap formation. Overexpression of EPLIN-β had a less disturbing effect on junctional dynamics compared to EPLIN-α, consistent with lower transmigratory rates. These findings are consistent with a previous reports examining barrier function and dynamics for the EPLIN isoforms^65,86^. In addition, other key players in actin dynamics as well as actin-independent molecules that interact with EPLIN, such as Rho GTPases, α-catenin, vinculin, cortactin, paxillin and α-catenin^38,41,61,62,86^, contribute to the TEM of neutrophils and other leukocytes. Furthermore, additional actin-independent effects ^101^ on TEM of leukocytes may play a role. Further studies exploring the interplay between these molecules and EPLIN isoforms will provide additional mechanistic insights. Together, EPLIN isoforms are critical for TNF-α-induced actin dynamics and transmigration, the overall regulation of transmigration rate, however, involves a complex interplay of multiple molecular factors.

EPLIN has been identified as a component of actin at transmigratory pores during transendothelial migration (TEM) in vivo. Further specific live cell imaging of fluorescently labelled EPLIN isoforms in HUVEC cultures showed that EPLIN-α can rapidly appear and disappear at transmigrating pores, forming a complete actin ring-like structure. In contrast, EPLIN-β showed an irregular distribution with lower dynamics at the transmigratory pore. Deletion of both EPLIN isoforms impaired actin ring and junction dynamics and reduced TEM. The actin ring is thought to support neutrophil passage between endothelial cells, while contractile actin bundles help to seal the transmigratory pore and prevent excessive increase in permeability (reviewed in^18^). The localisation of EPLIN-α to endothelial cell stress fibres suggests a role in controlling stress fibre turnover and remodelling, supported by FRAP analysis showing higher turnover rates for EPLIN-α compared to EPLIN-β in EA.hy KO cells, consistent with previous studies on HUVEC^65^. Furthermore, while the precise role of EPLIN-α in stress fibres needs to be further investigated, the significant reduction in stress fibres in EA.hy KO cells following selective EPLIN-α overexpression suggests that EPLIN-α counteracts the stress fibre-stabilising effect of EPLIN-β. The increased half-life of both EPLIN isoforms may be related to their enhanced integration in TNF-α-mediated stress fibre induction. While useful, EA.hy cells are less stable than HUVEC cultures due to their tumour-like characteristics, resulting in statistically non-significant TEM data between KO and EPLIN-α re-expressing cells, despite a clear trend. We hypothesize that this is due to the complex interplay between EPLIN isoforms, in particular the lack of a role for EPLIN-β in stress fibre formation and stabilisation.

#### Third, transmigratory gap closure is controlled by EPLIN-α

Once transmigration is complete, the transmigratory gap must be closed, with actin dynamics and membrane protrusions also playing an important role (reviewed in^5,32,102,103^). EPLIN-α plays an important role in the closure of transmigratory gaps after neurophil transmigration. The pore closure process and TNF-α induced junction dynamics involve membrane protrusions, similar to other Arp2/3 complex-driven processes such as (JAIL) or lateral lamellipodia which occur during cell migration or following proinflammatory stimulation and restore VE-cadherin-mediated cell adhesion^24,60,104^. EPLIN-α-positive structures were identified at branched actin-induced membrane protrusions during transmigration and pore closure, together with the Arp2/3 complex and LifeAct-EGFP. This is consistent with a role for Rac-WAVE/WASP-mediated control of the Arp2/3 complex in regulating transendothelial migration^24^. EPLIN-α controls Arp2/3 complex-induced JAIL by limiting these membrane proteins, thereby maintaining regulated protrusion dynamics. The JAIL-mediated and EPLIN-α-controlled mechanism is able to control barrier function through increased dynamics and close these gaps by restoring VE-cadherin adhesion. This process is similar to the EPLIN-α-mediated JAIL control observed in cell growth, wound healing and angiogenesis^54,55^. Understanding the entire dynamics of endothelial cells during inflammation may reveal additional key mechanisms involved in the transmigration process.

## Methods

All methods and experimental protocols were carried out in accordance with relevant guidelines and regulations.

### Antibodies and reagents

The following antibodies were used: rabbit anti-EPLIN (Bethyl-Biomol, Hamburg, Germany), goat anti-VE-cadherin (Santa Cruz, Heidelberg, Germany), mouse anti-ICAM-1 (BD Transduction Laboratories, Heidelberg, Germany), Secondary antibodies used in immunostaining were: Alexa 488 d-anti-rabbit, Alexa 488 d-anti-goat, Alexa 568 d-anti-goat, Alexa 568 d-anti-rabbit, Alexa 647 d-anti-mouse, and Alexa 633 d-anti-goat (BD, Transduction, Laboratories, Heidelberg, Germany). Antibodies used in flow cytometric analysis were: human CD54-FITC, human CD54-PE, mouse IgG2b-FITC, mouse IgG2bkappa-FITC (Immunotools, Friesoythe, Germany), human CD62E-FITC and human CD66b–FITC (ebioscience, Darmstadt, Germany). Phalloidin-TRITC was from Sigma-Aldrich (Deisenhofen, Germany) and DAPI from (Roche, Basel). All other chemicals used were from Sigma-Aldrich (Deisenhofen Germany).

### Preparation of mouse tissue to examine leukocyte transmigration in vivo after explantation

The LifeAct mice have previously been described (https://www.nature.com/articles/nmeth0310-168) and were kindly provided by Michael Sixt, Institute of Science and Technology, Austria. The mice were further bred at the Max Planck Institute for Molecular Biomedicine, Münster, Germany. Male LifeAct-GFP mice were irradiated (920 rad) and injected i.v. with 3–5 × 10^6^ bone marrow cells of WT C56Bl/6 mice. After six weeks of recovery, mice were injected intra-scrotally with 50 ng IL-1β for four hours. The mice were sacrificed by neck dislocation. Inflamed cremaster muscles were collected and fixed in 4% PFA/PBS on ice for one hour. Whole-mount tissue was permeabilized in 1% Triton X-100 for 2 min and stored in 2% BSA/PBS at 4 °C until immunostaining. Staining was performed on whole mount cremaster muscle samples. Incubation with primary antibodies was done in 2%BSA/PBS overnight at RT. After washing 3 times with 0.1% Triton X-100/PBS for 1-h, secondary antibodies were added in 2%BSA/PBS overnight at 4 °C. Next day, samples were washed again 3 times with 0.1% Triton X-100/PBS for 1 h each. At the end, samples were incubated with 1 µg/ml DAPI in PBS for 1 h. Samples were mounted in DAKO fluorescent mounting medium after subsequent washings and examined using LSM780/ELYRA at wave length as indicated (Carl Zeiss, Göttingen, Germany). All experiments were carried out as approved by the local state authorities (Office for the Protection of Nature and Environment of the State of North-Rhine-Westphalia, Germany = Landesamt für Natur, Umwelt und Verbraucherschutz Nordrhein-Westfalen (LANUV)). Authors complied with the ARRIVE guidelines and all methods were performed in accordance with the relevant guidelines and regulations of LANUV.

### Cell culture and coating of culture dishes

The use of human umbilical cords for cell isolation was approved by the ethics committee of the WWU Muenster (2009-537-f-S), and according to the principles outlined in the Declaration of Helsinki. Umbilical cords were collected from infants, with informed consent obtained from their legal guardians. Human umbilical veins (HUVEC) were perfused with PBS (#21600069, Thermo Fisher Scientific, Waltham, MA, USA) followed by treatment with 1 mg/ml^−1^ Collagenase (#C2-22, Biochrom, Berlin, Germany) diluted in PBS for 10 min at 37 °C in a water bath. The detached cells were collected and centrifuged at 200 × g. Cells were cultured in Endothelial growth medium (PromoCell, Heidelberg, Germany) supplemented with Endothelial Cell Growth Medium Supplement Mix and 1% penicillin/streptomycin (P/S) in tissue culture flasks. The first passage of isolated HUVEC were used for experiments, and seeded onto the respective crosslinked gelatin-coated culture supports. All procedures were performed according to^54^.

HL 60 promyelocytic cells were cultured in high glucose Dulbecco’s MEM (DMEM) supplemented with 10% FCS, 1% P/S and 1% sodium pyruvate (CCL-110 cells) or 1% Glutamine, and were differentiated with 1.3% DMSO/media and then used within 3–5 days.

EA.hy cell line was obtained from American Type Culture Collection (ATCC) and cultured in high glucose DMEM supplemented with 10% FCS, 1% P/S and 1% sodium pyruvate (CCL-110 cells) or 1% Glutamine in 5% CO^2^ and 37 °C incubator. These cells were seeded in cell density of 30 × 10^4^ cells/cm^2^ and passaged every 3 days.

### Coating of culture supports with crosslinked gelatin

Culture dishes and glass bottom dishes were coated with crosslinked gelatine^65^. Briefly, culture supports were coated with 0.5% procine skin gelatin (Siegma-Aldrich, Germany) for 1 h at RT, and subsequently exposed to 2.5% glutaraldehyde/PBS for 10 min at RT. After the solution was removed, 70% ethanol/H2O was added for 30 min, and subsequently washed with PBS for 5 times. Active aldehyde groups were neutralised using 2 mM Glycine/PBS overnight. Prior to cell seeding culture dishes or filters were washed 4 times with PBS.

### Activation of HUVEC with TNF-α and leukocyte adhesion assay

HUVEC monolayers were activated with recombinant human tumor necrosis factor (TNF-α) (Thermo Fischer Scientific, Darmstadt, Germany) in promo-cell medium at a final concentration of 50 ng/ml for 6 h.

### ICAM-1 cross linking and preparation of ICAM-1-coated polystyrene beads

To induce ICAM-1 clustering on activated HUVEC, mouse anti-ICAM-1 antibodies (1 µg/ml, 30 min) or unspecific mouse immune globulin (IgG) for control were added to the TNF-α activated HUVEC monolayer, followed by three times washing with PBS. Subsequently, an anti-mouse anti-body (1 µg/ml) was applied for 30 min followed by 2% formaldehyde/PBS fixation and immune labelled as indicated. All incubations were performed in starving medium.

Coating of polystyrene beads was performed as follows. One ml polystyrene beads (10 µm, Polyscience, Warrington, PA) were coated with ICAM-1 or IgG isotype as a control according to the manufacturer’s protocol. Briefly, beads were washed twice in PBS and incubated overnight with glutaraldehyde at room temperature under constant rotation and then washed with PBS. After incubation of the respective antibodies (1 g/2106 beads) for approximately 4 h, the beads were washed twice and subsequently incubated in 0.5 M ethanolamine in PBS for 30 min followed by a wash step in 10 mg/ml BSA in PBS for 30 min. The ICAM-1-coated beads were added to TNF-α stimulated HUVEC and incubated for 45 min. Subsequently, the cells with adherent beads were washed twice with cold PBS supplemented with 1 mM CaCl2 and 0.2 mM MgCl2, fixed with 2% formaldehyde in PBS and further processed for immune labeling.

### Isolation of human neutrophils

PMNs from human blood were isolated by density gradient centrifugation as described elsewhere^105^. 10 ml Histopaque 1077 were undercoated with 10 ml Histopaque 1119 (Sigma-Aldrich). Freshly collected EDTA blood (20 ml) was carefully applied on top and centrifuged at 550 g for 30 min. The phase containing granulocytes were collected, washed twice in wash buffer (HBSS − / − , 25 mM Hepes, pH 7.3, and 10% FCS). PMNs were used for transmigration assays directly after isolation as indicated.

### Leukocyte transmigration assays in vitro 

1 × 10^6^ freshly isolated neutrophils in HEPES medium were perfused over HUVEC monolayers at 1.0 dyn/cm^2^ at 37 °C and under 5% CO_2_. Phase contrast live cell imaging was performed using an automated Observer microscope (Carl Zeiss, Göttingen, Germany) (10 × objective). Time-lapse images were acquired in four randomly selected fields of the flow chamber at 20-s intervals. Neutrophil adhesion was then counted exactly 6 min after neutrophil addition. The total number of migrated neutrophils was counted and then plotted on a graph. Transmigrated neutrophils were distinguished from adherent cells by their change in appearance in phase-contrast microscopy. After transmigration, the cells spread under the endothelium and gradually lose their spherical structure with high contrast. ZEN software (Carl Zeiss, Göttingen, Germany) was used for image acquisition and analysis as indicated. Statistical analysis was performed using GraphPad Prism software with an unpaired t-test after normality was confirmed by the Shapiro–Wilk test. Approximately 1.000 cells were counted from four independent experiments.

### Downregulation of EPLIN in HUVEC by siRNA approach

Downregulation of EPLIN in HUVEC cultures was performed by the siRNA (siEPLIN) approach using the Magnet-Assisted Transfec-tion protocol (MATra from Promokine, Heidelberg, Germany). Briefly, cultures were washed with antibiotic- and serum-free Promo-cell medium. ON-TARGET plus NON-targeting siRNA#1 (sequence: UGGUUUACAUGUCGACUAA) or ON-TARGET plus SMART pool siRNA, LIMA1 (sequences: GCUUAAACAUUACGACUGA, UGUUAGUGUUAGCGAGCCA, GAAGAAGCUAAGACGAUCU, AGG UUAAGAGUGAGGUUCA) (Dharmacon, Lafayette, USA) were diluted in serum-free medium together with the MATra siRNA-reagent (Promokine) and incubated for 20 min. Solutions were added to the cells and exposed to the magnetic field for 15 min at 37 °C with 5%CO_2_ and further cultured for 4–6 h. After medium exchange, the cells were cultured, usually up to 72 h, until the proteins were downregulated. This procedure was performed as described elsewhere^65^.

### Virus particle production and lentivirus mediated gene transduction

Plasmid constructs for EPLIN-α or β EGFP/mCherry, EGFP-p20 and LifeAct-EGFP were prepared as described elsewhere^54^. Briefly, to produce virus particles, HEK293T cells were cultured in DMEM (high glucose) medium in 15-cm cell culture dishes. Accordingly, pFUGW vectors carrying the gene of interest, the packaging vectors (pCMV-ΔR8.74), and the VSV glycoprotein-carrying vector (pMD2G) were transduced to these cells. Plasmids (pFUGW-gene of interest (about 23 μg), pCMV-ΔR8.74 (23 μg), and pMD2G (11,5 μg)) were dissolved in dissolving solution of 1.725 ml DMEM medium. The transfection solution containing 124,2 μl of the transfection reagent PEI (1 mg/ml) dissolved in 1600.8 μl DMEM-/ − was prepared. Solutions were kept at RT for 20 min before being mixed together and adding to HEK293T cells. 24 h later, the medium was changed with complete DMEM. The medium was then collected 24 h later, and the supernatant filtered through 0.45-μm filters, followed by ultracentrifugation (1.5 h at 25.000 rpm; 4 °C). 150 μl PBS containing 1% BSA was prepared for virus particle resuspension; then the virus suspension was preserved at -80° C until use.

Prior to gene transduction into endothelial cells the respective virus titers were determined by bioassay using HUVEC cultures followed by Western blot analyses. The desired virus titer was then applied to HUVEC cultures that were seeded at a density of 2 × 10^4^/cm^2^ and cultured overnight. Next day, the required amount of virus particles was resuspended in Promocell medium. Following one hour of incubation, 1 ml Promocell complete medium was added to cells. The medium was replaced with fresh medium next day. Expression of the respective proteins occurred between 2 and 3 days later^54^.

### Fluorescence live-cell imaging and image acquisition

Fluorescence live-cell imaging of HUVEC cultures expressing fusion proteins was performed by Spinning Disk Microscopy (SpDC) using an automated Observer (Carl Zeiss, Göttingen, Germany) at 37 °C with 5% CO_2_ using a culture dish perfusion system containing a 1 cm in diameter glass-bottomed 35-mm culture petri dishes. Image acquisition was performed using Plan Apo 1.3 oil 40 × objective lenses (Carl Zeiss, Göttingen, Germany). EGFP was excited with a 488-nm laser line (Argon laser) and emission was recorded using a 38 HE Green filter. mCherry was excited with a 543-nm laser line and detected through a 43 HE DsRed filters. The ZEN software (Carl Zeiss, Göttingen, Germany) was used for image acquisition and analyses, as indicated. EPLIN-α-EGFP intensity was measured from the onset of transendothelial migration (TEM) at 00:00 until the gap was completely closed at 07:10 using line scan analysis in Fiji. Colocalisation of EPLIN-α-mCherry and LifeAct-EGFP was quantified using the Coloc-2 plug-in in Fiji.

### Immune labelling and fluorescence microscopy of cultured cells

For immune labelling, cultured cells were fixed with freshly prepared 2% paraformaldehyde dissolved in PBS for 10 min at room temeprature. The cells were then washed three times for 5 min each, using PBS containing 1% BSA (PBS/BSA). Cell membranes were made permeable with 0.1%Triton X-100 in PBS for 10 min at 4 °C, washed again three times with PBS/BSA, followed by incubation with the respective antibody for 1 h at RT or overnight at 4 °C as indicated, washed again and exposed to appropriate secondary antibodies. Cultures were mounted in Dakofluorescence mounting medium and evaluated by either LSM or SIM using LSM 780 supplemented with ELYRA super resolution module (Carl Zeiss, Göttingen, Germany). The fluorescence intensity of the blue channel has been increased to improve the visibility of the corresponding labels.

The expression of cell adhesion molecules in HUVEC was determined by indirect immunolabeling followed by flow cytometry (FACScan, Becton Dickinson). For this purpose, treated HUVEC were labeled with the primary antibodies, washed twice with prewarmed PBS, and then detached with Accutase for 5 min. Subsequently, the cells were centrifuged at 1000 rpm and then incubated with FITC- or PE-conjugated secondary antibodies. Isotype-specific antibodies were used as controls. Washing steps with PBS were followed by flow cytometry.

### Western blot

Prior to Western blot analyses the total protein concentration of SDS-solubilized or Triton-extracted samples was determined by the amido-black method^106^. Same amounts of protein were loaded on each lane. Subsequently, western blotting was performed using standard protocols as described elsewhere^54^. After protein transfer, membranes were incubated with primary antibodies diluted in 5% skimmed milk buffer over night at 4 °C. Then membranes were washed three times with PBST, and were incubated with secondary antibodies at RT for one hour and three additional, subsequent washes. Bands were detected using LI-COR Infrared system including the odyssey software package (LI-COR Biotechnology, Bad Homburg, Germany).

### Elasticity measurements

Force mapping was performed in living HUVEC cultured in Promocell-medium stabilized with 10 mM Hepes at 37 °C temperature using a BioScope Catalyst™-AFM (Bruker Nano Surfaces, Santa Barbara, California, USA) in closed-loop mode with ramp size of 2 µm, max. force of 1nN and a tip velocity of 2.55 µm/s. Measurements were taken at the highest point of the cell (core/perinuclear region) to assess overall cell stiffness. Each force map contained 16 × 16 force-distance cycles over an area of 150 × 150 µm. Three independent dishes of EPLIN-depleted and control cells respectively were measured with recording of 3 to 4 force maps on each dish. A Large-Radius-Bio-Probe SAA-SPH-5UM from Bruker (Camarillo, USA) (https://www.brukerafmprobes.com/p-4058-saa-sph-5um.aspx) with a tip radius of 3.46 µm was used. Spring constant of the cantilever (0.1913 N/m) was determined with an interferometer (OFV-551, Polytec, Waldbronn, Germany), and deflection sensitivity was adjusted according to the SNAP procedure^80^. The analysis of the force–indentation curves was performed with PUNIAS software (http://punias.free.fr/) using the linearized Hertz model^77^.

### Laser ablation experiments

Nanoablation was performed in Lifeact-EGFP expressing or EGFP-EPLIN-isoform-expressing EA.hy cell lines. An Imager M2 microscope (Carl Zeiss, Oberkochen, Germany) with a Yokogawa CSU-X1 spinning disk and sCMOS ORCA Flash 4.0LT system plus a Zeiss alpha Plan-Apochromat 100x/1.46 Oil DIC (UV) VIS-IR objective were used. For ablation experiments an ultraviolet-laser ablation system UGA-42 Firefly equipped with a DPSL-355/14 (Rapp OptoElectronics, Wedel, Germany) was used. Prior to acquisition, the laser was calibrated and the power was adjusted to 2% for all experiments. Time-lapse images were recorded every 200 ms before and after laser cut. The the recoil velocity and retracted distance were analyzed by using the line scan and kymograph tools provided by Fiji ImageJ.

The elastic stiffness tau (τ) was calculated based on the Kelvin-Voigt model^107–109^. Data were fitted to the following exponential function using MATLAB’s Curve Fitting tool (MathWorks) to determine tau (τ):

$$L\left( t \right)D\left( {1 - e^{{\frac{t}{\tau }}} } \right)$$

L(t) describes the displacement over time t and D indicates the force/elasticity ratio. Higher tension is indicated by decreased τ levels and less tension by increased τ.

### Determination of transendothelial electrical resistance (TER) from Impedance spectroscopy measurements

TER of growing EA.hy cell lines was determined using trans-well filters (ThinCerts™- TC inserts, 3-µm pore size (Sigma-Aldrich, Taufkirchen, Germany). Prior to cell seeding of 5 × 10^4^ cells/filter, filters were coated with fibronectin (3 µg/cm^2^, 1 h), and subsequently, cells were allowed to settle for 24 h. EA.hy seeded filters were inserted into metal-filter supports, designed for Impedance spectroscopy (ISP) measuements (MOS-Technologies, Telgte, Germany). ISP measurements were continuously recorded during cell growth. TER was determined from impedance spectroscopy measurements using analytical software (MOS-Technologies, Telgte, Germany).

### Fluorescence recovery after photobleachng (FRAP)

EPLIN-depleted EA.hy cells expressing either EPLIN-α-EGFP or EPLIN-β-EGFP were used for FRAP analysis. Cells were seeded at a density of 2 × 10^4 cells/cm^2 and cultured for 48 h prior to the experiment. FRAP analysis was performed using a Zeiss 880 Elyra laser scanning microscope. Pre-bleach and post-bleach images were acquired after excitation with a 488 nm laser at 1% power and emission in 539 nm using a Plan Apochromat 63 × objective/1.4 oil DIC M27 immersion objective. Before bleaching, 3 images were acquired. Circular regions of interest (ROIs) with a radius of 3 μm were defined and bleached using 100% power of the 405 nm, 458 nm, 488 nm and 561 nm laser lines. Fluorescence recovery was monitored continuously for 40 s after bleaching. The fluorescence intensity of the ROIs was then analysed using ZEN software. Specifically, after background subtraction, the fluorescence intensity within the ROI was normalised to the fluorescence intensity prior to bleaching. The resulting recovery curve was fitted with an exponential function.

### CRISPR/Cas9-mediated genome editing

EA.hy926 cells lacking EPLIN-α and EPLIN-β genes were generated using CRISPR/Cas9 technology essentially as described before^110,111^. The gene giving rise to both isoforms was disrupted by targeting either exon 4 (for Eplin KO #26) or exon 8 (for Eplin KO #6) with sgRNA sequences AGGTGAACCAACTCAAACTA or TGAGGTCTGCATCACCCATC. Guide sequences were cloned into the pSpCas9 (BB)-2A-Puro vector as recommended (Addgene plasmid ID: 48139). EA.hy926 cells grown in 6-well plates were transfected overnight with 1-3µg of respective plasmids and selected for 48 h with 1 µg/ml puromycin. After selection, cells were cultivated in regular medium (with medium change every 4 days) for around 2–3 weeks until they were sub-confluent. Next, cells were extensively diluted and after two weeks, cell colonies picked to obtain single-cell-derived clones. Approximately 3–5 weeks later, cell clones were transferred from 96- and 24- onto 6 well-plates, and cell lysates prepared from parallel cell fractions. Cell clones were screened for the absence of Eplin expression by Western blotting using an anti-Eplin antibody (Bethyl-Biomol Cat#A300-225A). Next, selected clones were subjected to genomic DNA preparation and modified regions within exons 4 and 8 amplified using PCR with the following primers: fw: 5’-ACAGAGCAGACCATCCTCCT-3’ and rev: 5 ‘-ACTGACCACATATTCGCCTCT-3’ (exon 4) and fw: 5’-TCACTAGTCAGTGGGTTTTGCT-3’ and rev: 5 ‘-TCTGTCCTGGCAGCTGTTAC-3’ (exon 8). To confirm the lack of respective wild-type alleles in modified clones, amplified DNA regions were then analyzed by sequencing using aforementioned fw-primers and further analyzed by TIDE (Tracking of Indels by Decomposition (http://tide.nki.nl), as described in ^112^.

### Re-expression of EPLIN-α EGFP or EPLIN-β EGFP EA.hy Eplin-KO cells

Eplin-KO EA.hy cells were seeded on 6-well plates and next day were incubated with lenti virus particles containing EPLIN-α-EGFP or EPLIN-β-EGFP (3μl/cm^2^) and cultured for 48 h. Then expression of EGFP was evaluated by fluorescent microscope. Viral infected cells were further cultured and expanded. EGFP positive cells were sorted by FACS-Aria III cell sorter (Becton–Dickinson). This way, stable EPLIN-α-EGFP or EPLIN-β-EGFP expressing Eplin-KO EA.hy cell lines were established and applied for further experimentations.

### Transmigration of neutrophils in trans-well filter assay

To analyze the effects of EPLIN-α or EPLIN-β on the transmigration of PMN, 2 × 10^4^ EPLIN-KO EA.hy926, cells re-expressing EPLIN-α-EGFP or EPLIN-β-EGFP were seeded onto fibronectin-coated (3 µg/cm^2^, 1 h) trans-well filters (6.5 mm, 5-µm pore size; Corning), grown to confluence, and stimulated with 50 ng recombinant human TNF-α (PeproTech) 6 h prior to the assay. Freshly isolated neutrophils (2 × 10^5^) were added to the cell monolayer, and allowed to transmigrate towards bottom wells containing chemokine interleukin-8 (IL-8; R&D Systems) for 30 min. Transmigrated PMNs were counted using an automated cell counter.

### Statistics

Statistical analyses were performed using GraphPad software (La Jolla, CA) available as an online calculator. The Shapiro–Wilk test was performed to assess the normality of the sample distribution. Student’s *t* was used for comparing the data between two groups. Differences between datasets were considered to be statistically significant with *p* < 0.05.

## Supplementary Information

Supplementary Video 1.

Supplementary Video 2.

Supplementary Information 1.

Supplementary Information 2.

## Data Availability

The datasets used and/or analysed during the current study available from the corresponding author on reasonable request.
