# Supplementary material for: A specific role for endothelial EPLIN-isoform-regulated actin dynamics in neutrophil transmigration
Source: Sci Rep. 2025 May 5;15:15698. doi: 10.1038/s41598-025-98192-9 (PMC12053001; doi:10.1038/s41598-025-98192-9)
Supplement: Supplementary file 3 — Supplementary Information 1. [file 41598_2025_98192_MOESM3_ESM.pdf]

Supplementary figure 1A-Aldirawi, Ghanbari et al.

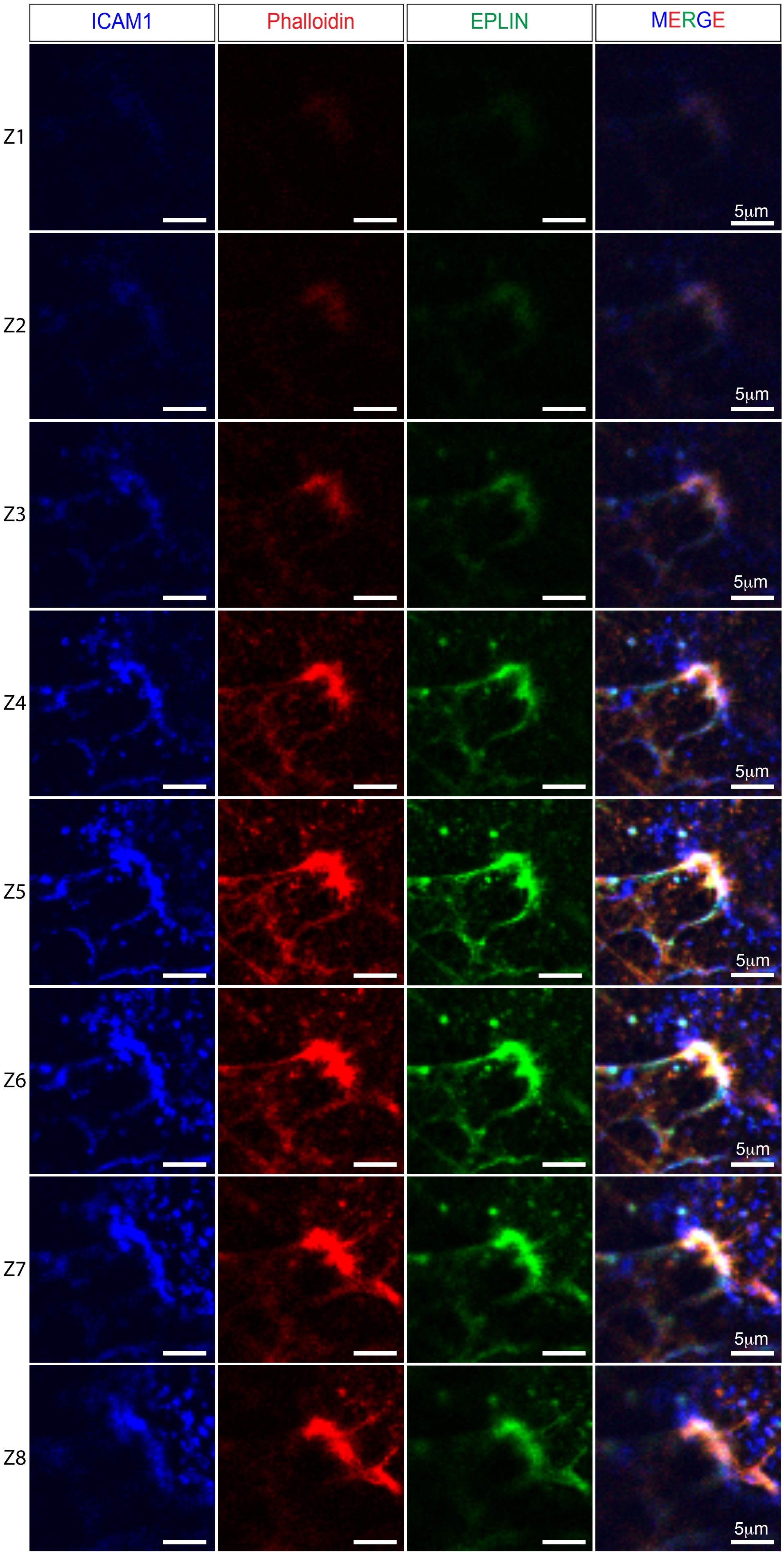

**Supplementary figure 1B-Aldirawi, Ghanbari et al.**

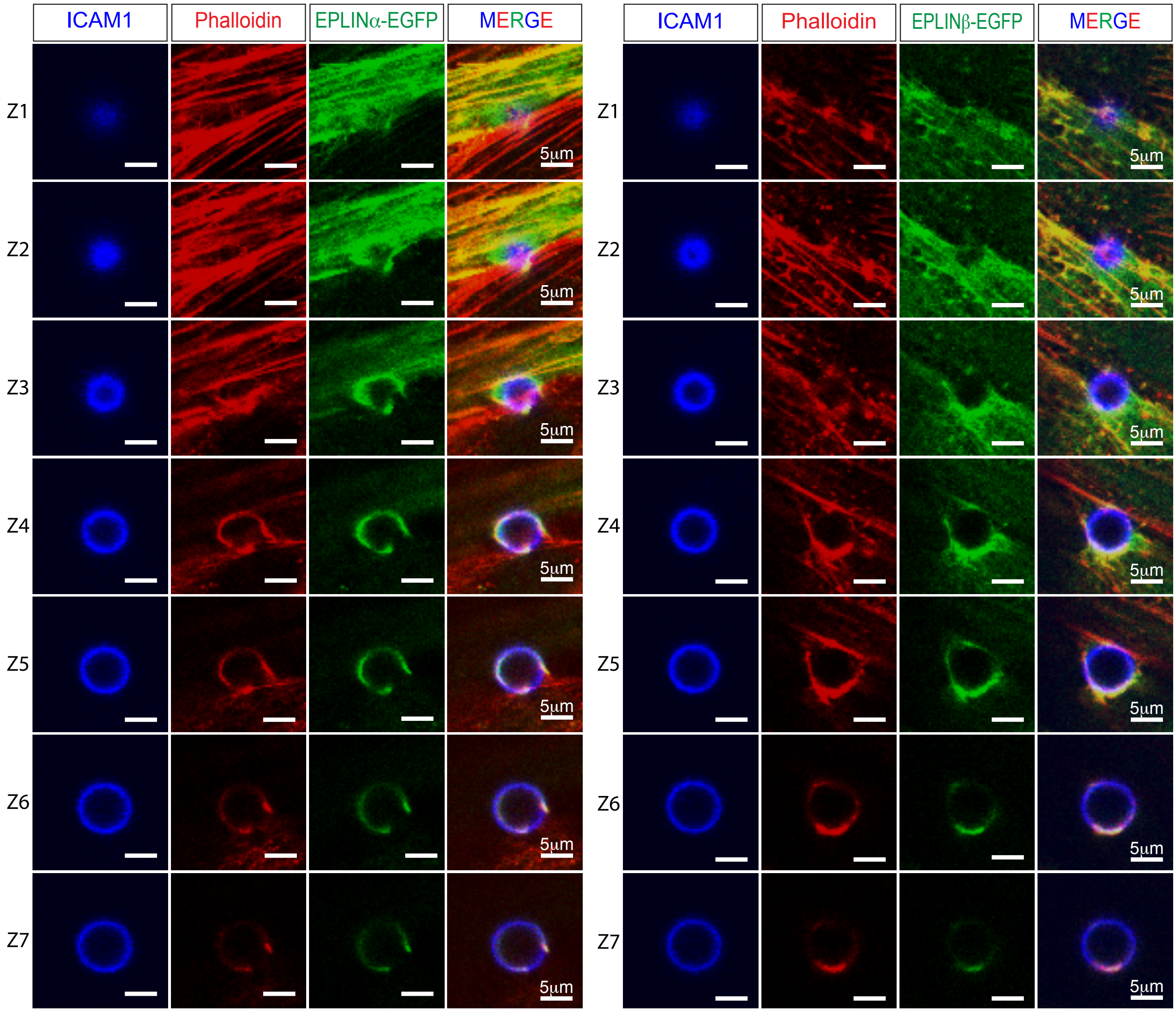

**Supplementary figure 1C-Aldirawi, Ghanbari et al.**

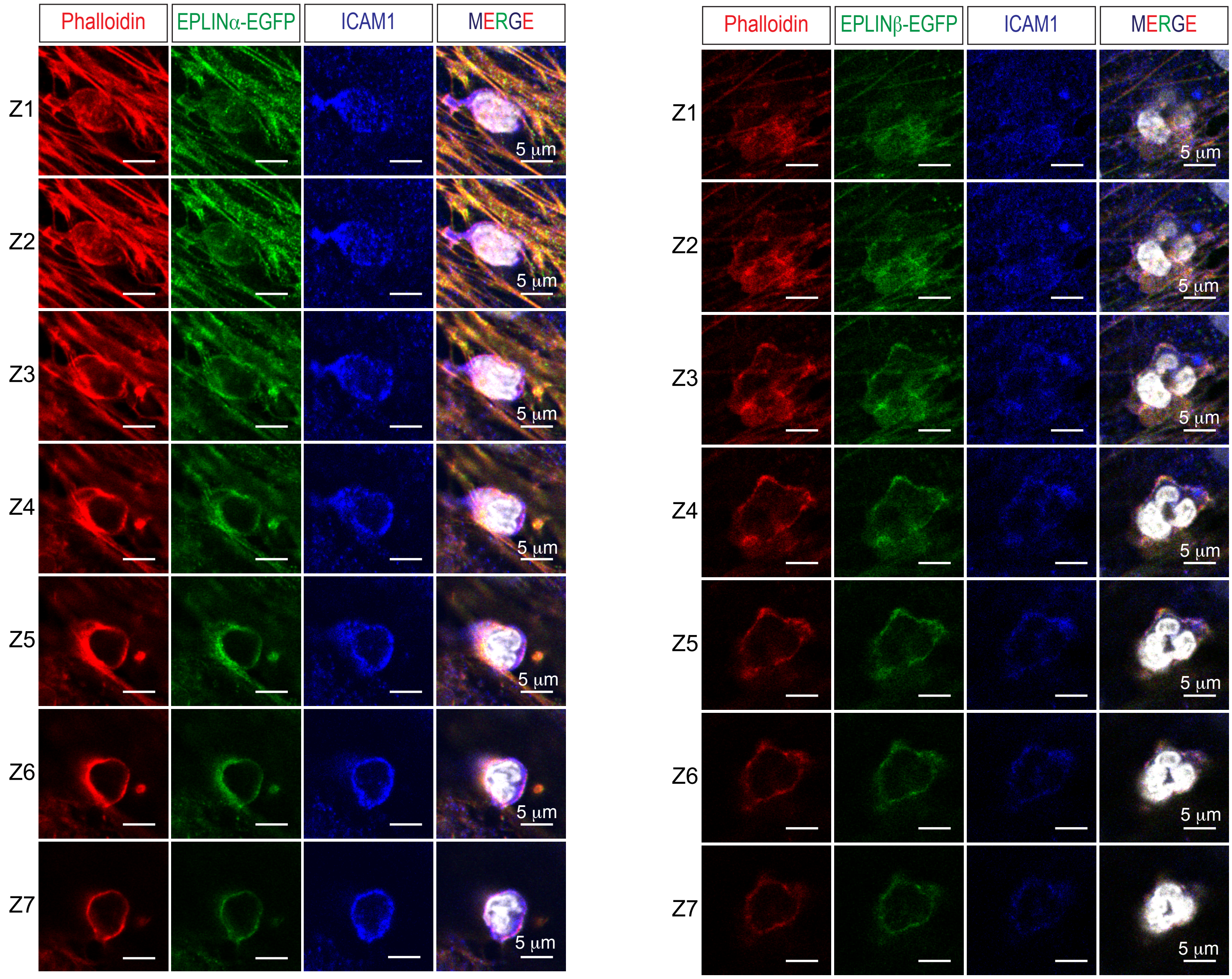

**Supplementary figure 1D-Aldirawi, Ghanbari et al.**

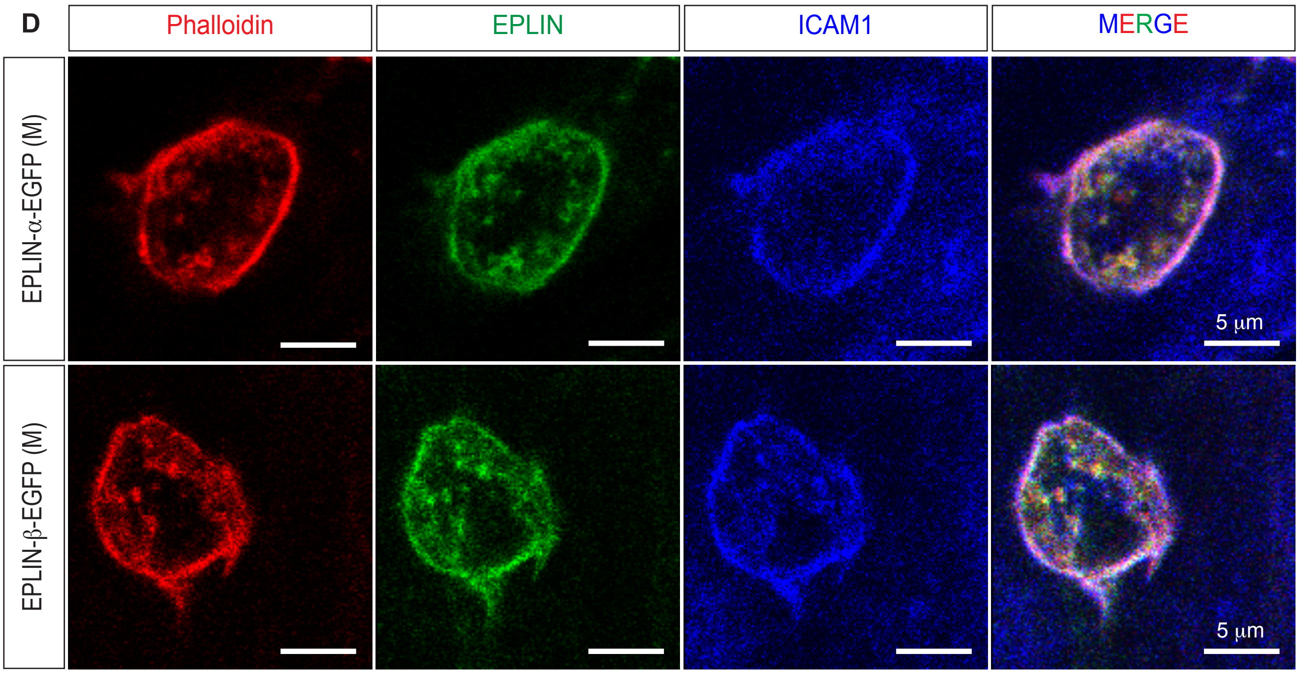


Supplementary Figure 1. EPLIN-α-EGFP and EPLIN-β-EGFP colocalized with ICAM. (**A**) Z-stacks images of antibody-induced ICAM clusters in TNF-α-activated HUVECs. (**B**) Z-stack images of TNF-α-activated and EPLIN-α-EGFP or EPLIN-β-EGFP expressing HUVEC cultures incubated with anti-ICAM-coated beads or (**C**) human neutrophils. (**D**) Confocal microscopy of TNF-α activated HUVEC cultures moderately expressing either EPLIN-α-EGFP (M) or EPLIN-β-EGFP (M) after exposure to the promyeloid leukemia HL60 cell line. Both proteins are components of actin filaments (Phalloidin-TRITC label) surrounding transmigrating HL 60 cells and colocalize with ICAM-1 clusters.

**Supplementary figure 2-Aldirawi, Ghanbari et al.**

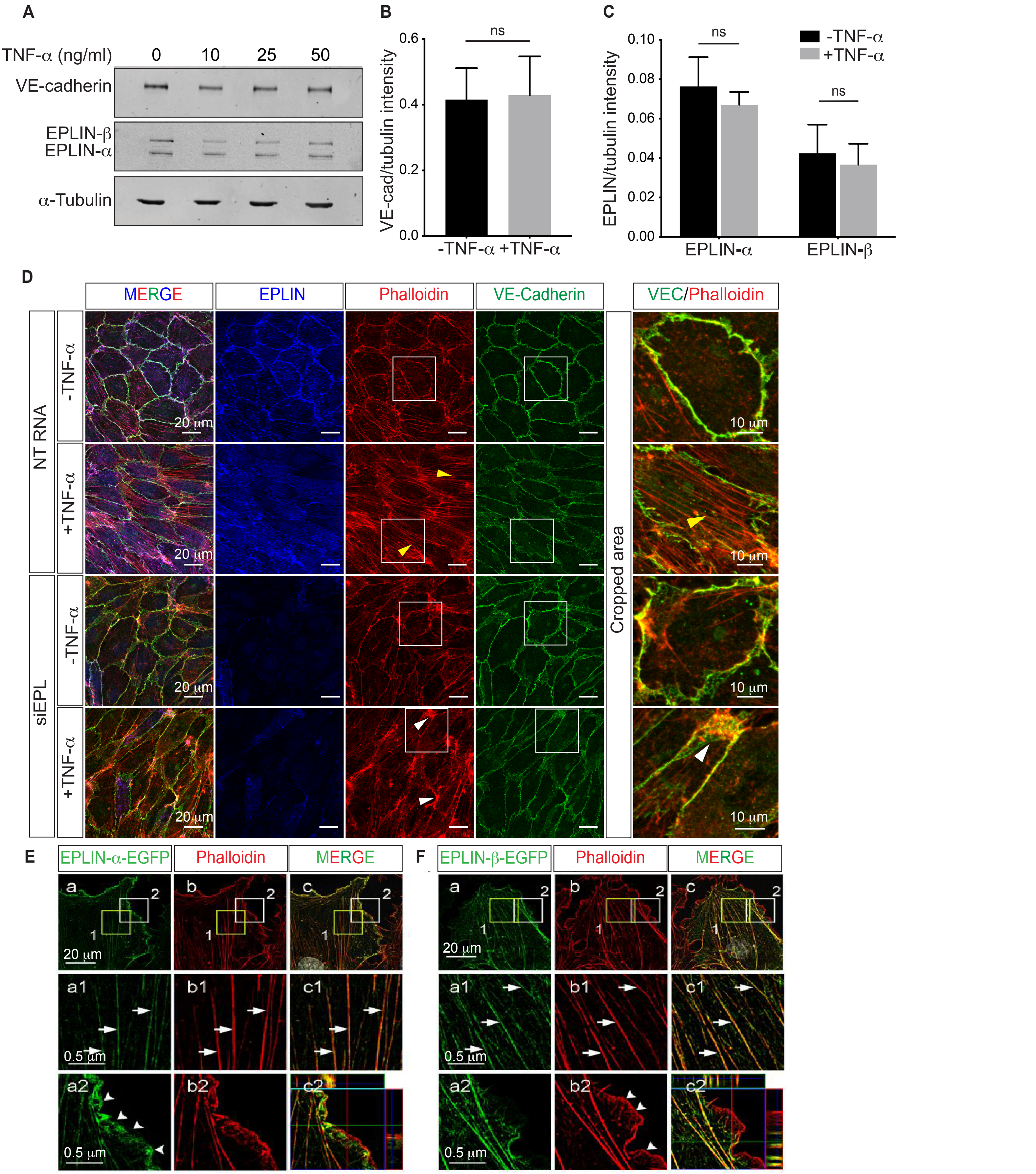


Supplementary Figure 2. (**A**) TNF-α does not significantly modulate the expression levels of VE-cadherin and EPLIN-α or EPLIN-β in confluent HUVEC cultures. HUVEC were treated with different concentrations of TNF-α for 6 hours followed by Western blot. α-tubulin served as an internal loading control. Full immunoblots are shown in supplementary fig.S7. (**B**,**C**) Quantitation of VE-cadherin and EPLIN isoforms intensity through one-way ANOVA of three independent experiments. Comparable levels are found in control and TNF-α-treated (50ng/ml) cells. Error bars represent ± SEM. (**D**) Localization of VE-cadherin and ICAM1 in TNF-α treated EPLIN-depleted HUVEC cultures. Confluent HUVEC cultures were either treated with NTRNA or siEPL for 48 h, then activated with TNF-α (50ng/ml) for 6 h and indirectly immunolabeled with anti-VE-cadherin (green) and anti-EPLIN (blue). Phalloidin-TRITC (red) was used to label actin filaments. Yellow arrowheads show the stress fibres and the white arrow heads show the abbortive JAIL.(**E-F**) SIM pictures of sparse HUVEC expressing EPLIN-α-EGFP or EPLIN-β-EGFP were fixed and stained with phalloidin-TRITC. Yellow and white boxes (a-c) indicate areas of higher magnifications (a1-c2). Large arrow heads point to separated actin and EPLIN at protrusions, white arrows to actin stress fibres spotted with EPLIN clusters.

**Supplementary figure 3-Aldirawi, Ghanbari et al.**

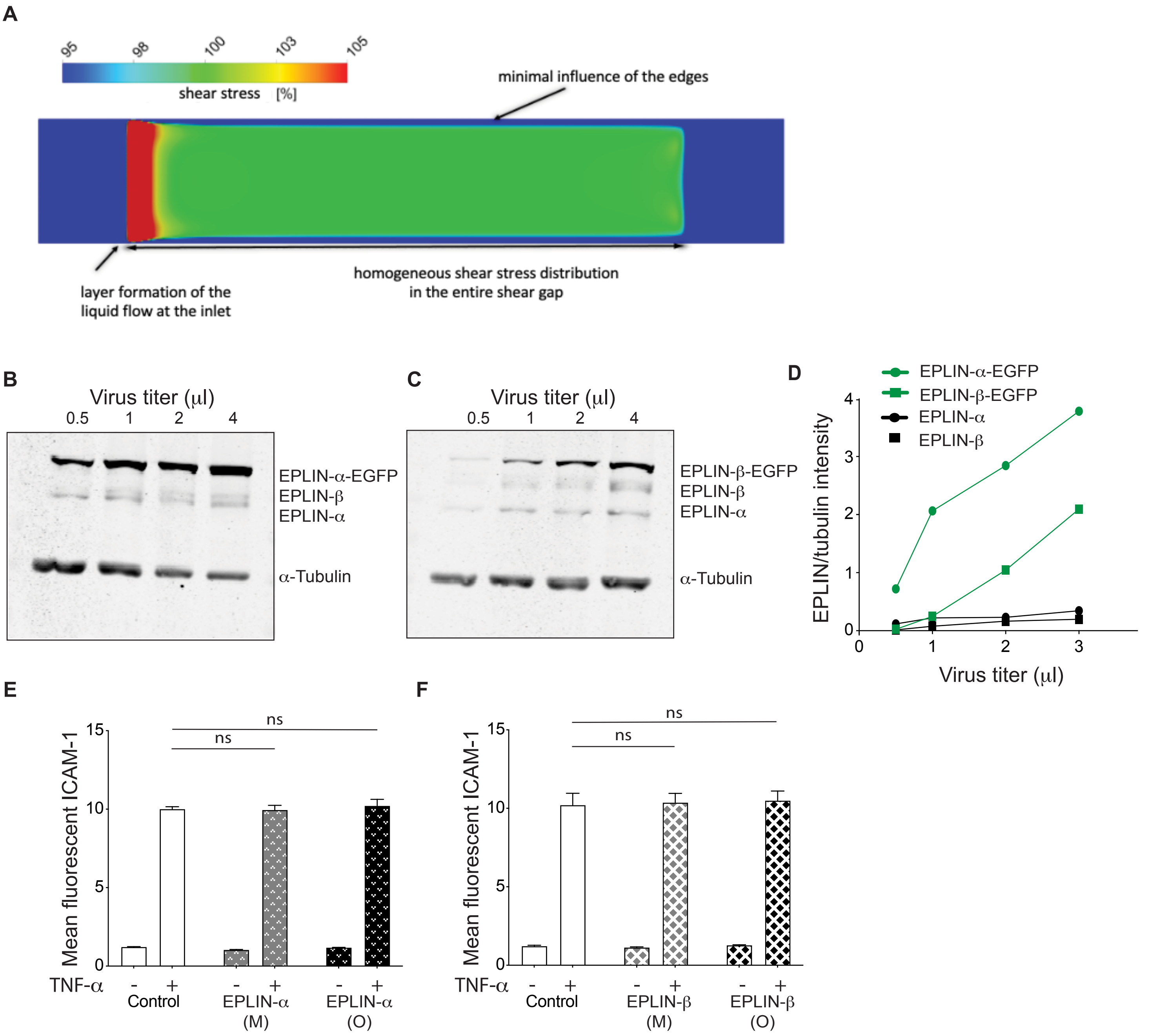


**Supplementary Figure 3.** (**A**) Numerical simulation of the flow profiles in the flow chamber used for the experiments shows a very homogeneous flow pattern as demonstrated by false color. (**B-C**) Dose-dependent analysis of the expression level of fluorescently labeled EPLIN isoforms in HUVEC as indicated, analyzed by western blotting using anti-EPLIN and anti-α-tubulin as internal controls. Full immunoblots are shown in supplementary fig.S7. (**D**) Quantification of protein bands, with average intensity plotted against viral titers. (**E**,**F**) Activation of HUVEC by TNF-α after moderate or overexpression of EPLIN isoforms remained comparable by measuring by ICAM-1 expression through FACS analysis. ns= not significant.

**Supplementary figure 4-Aldirawi, Ghanbari et al.**

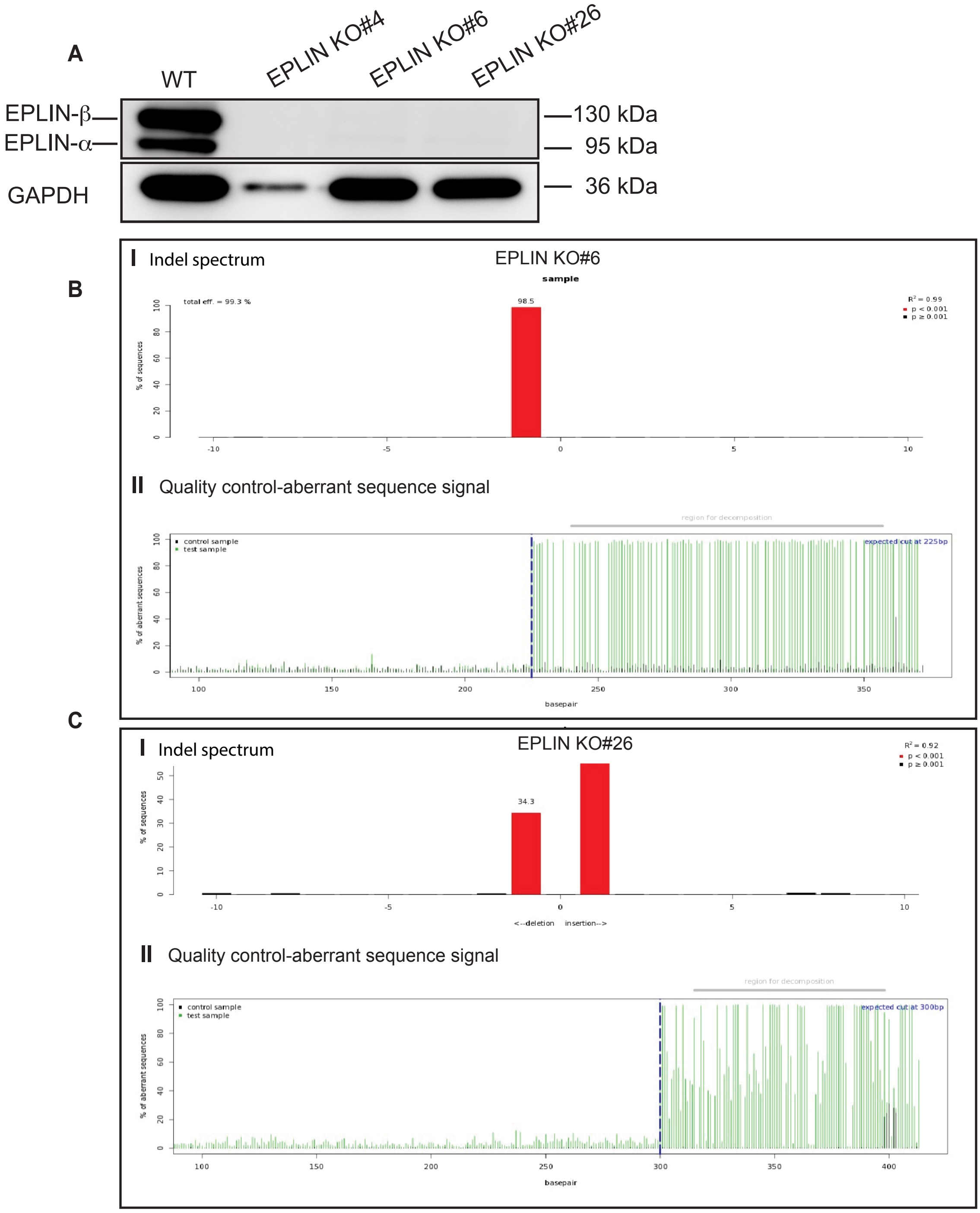


**Supplementary Figure 4.** Generation of EA.hy926 EPLIN KO cells.
(**A**) EA.hy926 WT and EA.hy926 EPLIN KO clones after CRISPR/Cas9-targeting (see methods) were lysed and analyzed by Western blotting. Blots were probed with anti-EPLIN and anti-GAPDH antibodies as loading control. full blots are shown in supplementary fig.S7 Blot showing promising EA.hy926 EPLIN KO clones, with clones #6 and #26 selected for future analyses. (**B**-**C**) Assessment of genome editing in EPLIN KO #6 and EPLIN KO #26 clones by TIDE (Tracking of Indels by Decomposition, http://tide.nki.nl). (**I**) Respective quantification of editing efficacy showing the spectrum of indels and their frequencies in EPLIN KO samples. Note that no wild-type allele was detected (0) in the respective genomic regions in EPLIN KO samples. (II) Visualization of aberrant sequence signals for each clone; WT and KO samples are displayed in black and green, respectively. Blue dotted lines indicate expected cutting sites. Gray horizontal bars display the regions used for decomposition. R2 represents the goodness of fit and the P-value is calculated by a two-tailed t-test of the variance–covari-

**Supplementary figure 5-Aldirawi, Ghanbari et al.**

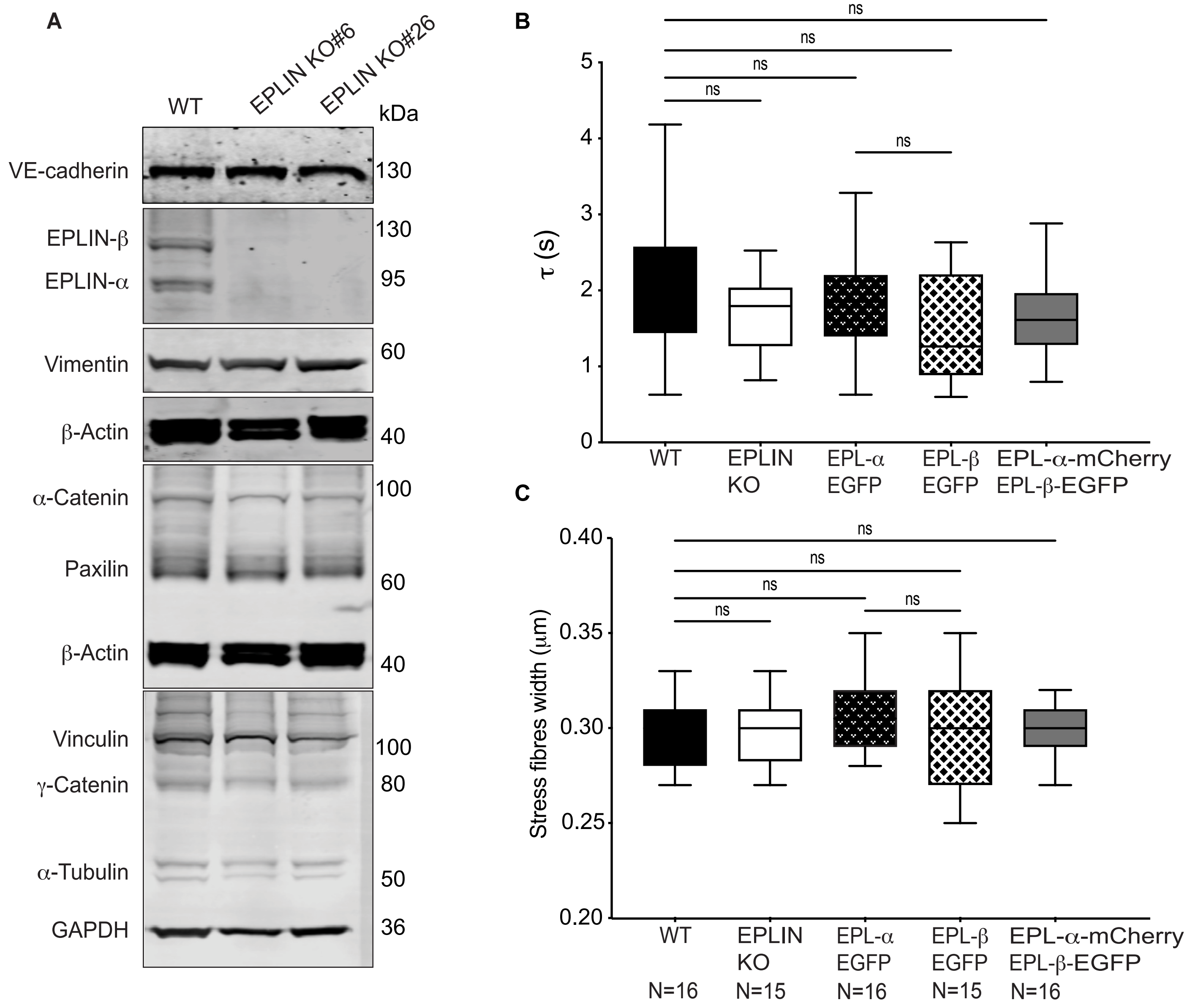


**Supplementary Figure 5.** (**A**) Characterization of CRISPR/Cas-mediated EPLIN knockout in EA.hy endothelial cell lines. Knockout of EPLIN isoforms did not significantly affect the expression or distribution of VE-cadherin and cytoskeleton- and cytoskeleton-associated proteins as indicated. Full immunoblots are shown in supplementary fig.S7. (**B** and **C**) Laser ablation of actin stress fibres in EAhy-EPLIN-KO and isoform reexpressing EAhy cell lines. Laser ablation of stress fibres was performed in EAhy-WT cell lines as well as in EAhy-EPLIN-KO and selectively EPLIN α-EGFP or EPLIN-β-EGFP or EPLIN-α-mCherry+EPLIN-β-EGFP expressing EAhy cells to investigate the impact of EPLIN isoforms on stress fibres contractility. Labelled actin stress fibres (EPLIN-KO and EAhy-WT cells transduced with LifeAct-GFP) were cut using a 355nm laser system. Combined with a spinning disc microscope, the displacement of the separated actin ends can be tracked and displacement velocity can be determined. Stress fibres can be understood in its grossest simplification as a Kelvin-Body (Kumar et al., 2006). Using this mathematical-physical model, it is possible to determine the elastic stiffness (τ), which can be used as a measure of tension (contractility) (for further description see Zulueta-Coarasa and Fernandez-Gonzalez, 2015). (**B**) Determination of the elastic stiffness (τ) in EAhy-EPLIN-KO and isoform reexpressing cell lines. Box and whisker plots show values of 3 independent experiments (n= number of laser cuts per cell line). (**C**) The measured widths of the dissected stress fibres are shown in the box and whisker plots (values of 3 independent experiments; n= number of stress fibres per cell line). ns = not significant.

Supplementary figure 6-Aldirawi, Ghanbari et al.

## Figure 5A

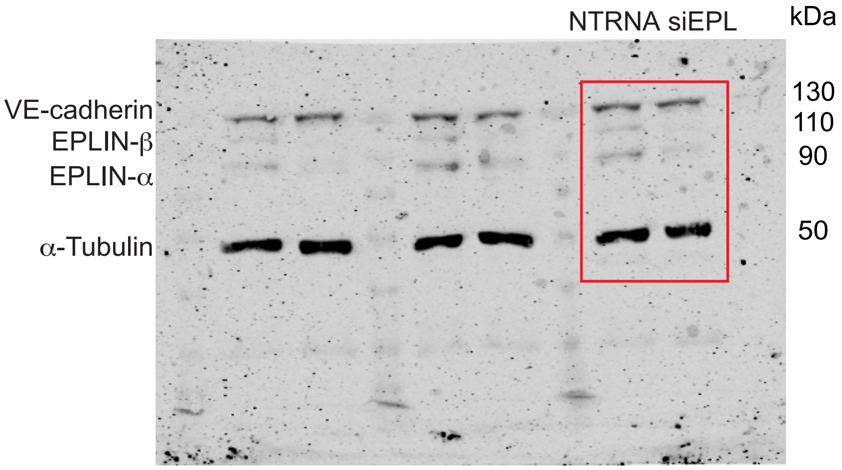


## Figure 6B

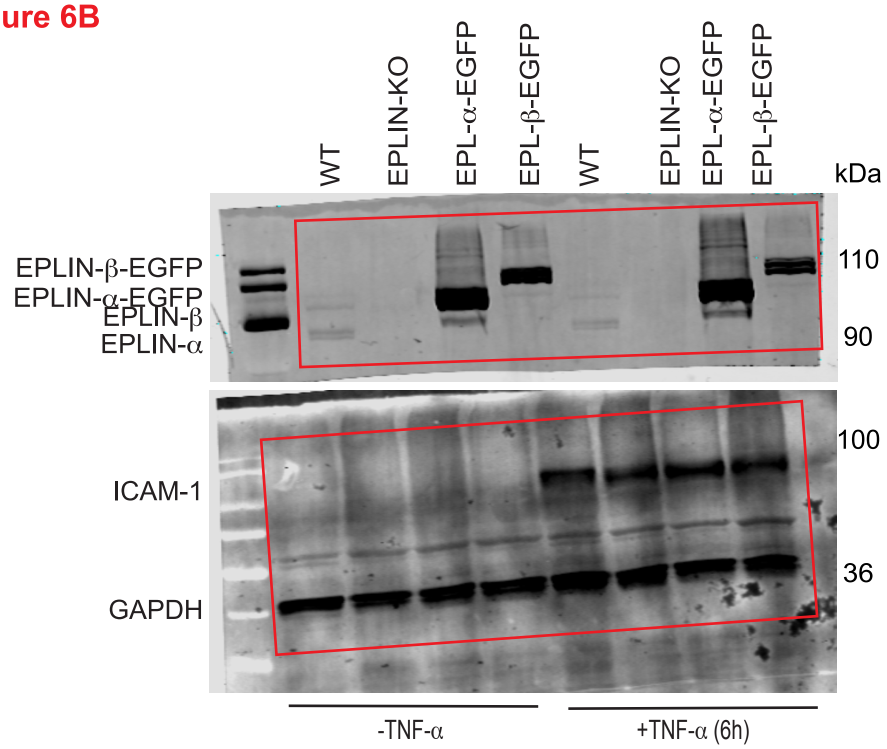


Supplementary Figure 6. Full immunoblots The area of the original blots shown in related main figures are framed in red

**Supplementary figure 7-Aldirawi, Ghanbari et al.**

## Sup fig. 2A

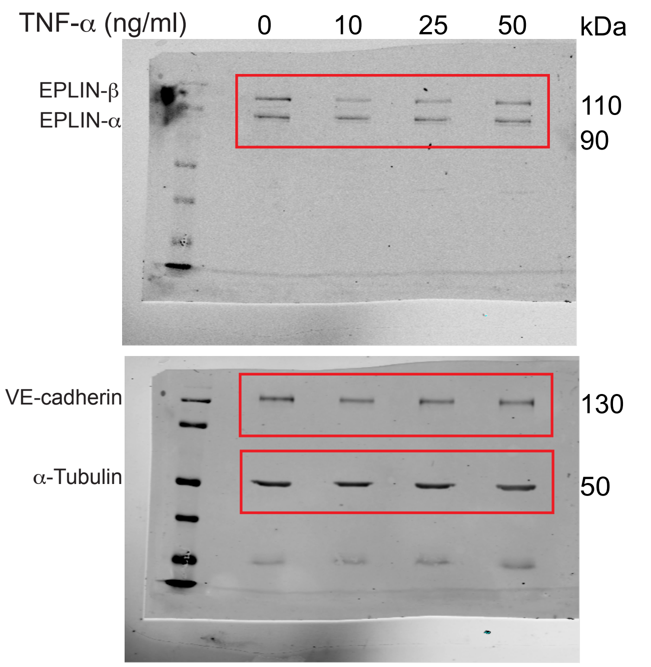


## Sup fig. 3B-C

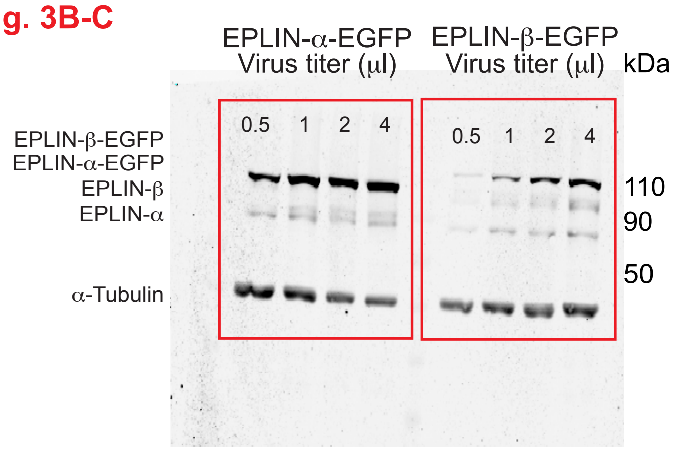


## Sup fig. 4A

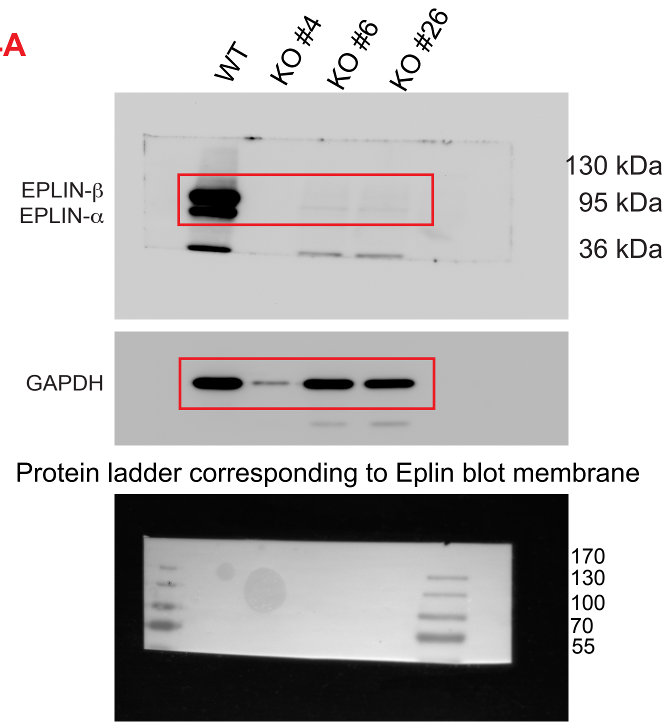


## Sup fig. 5A

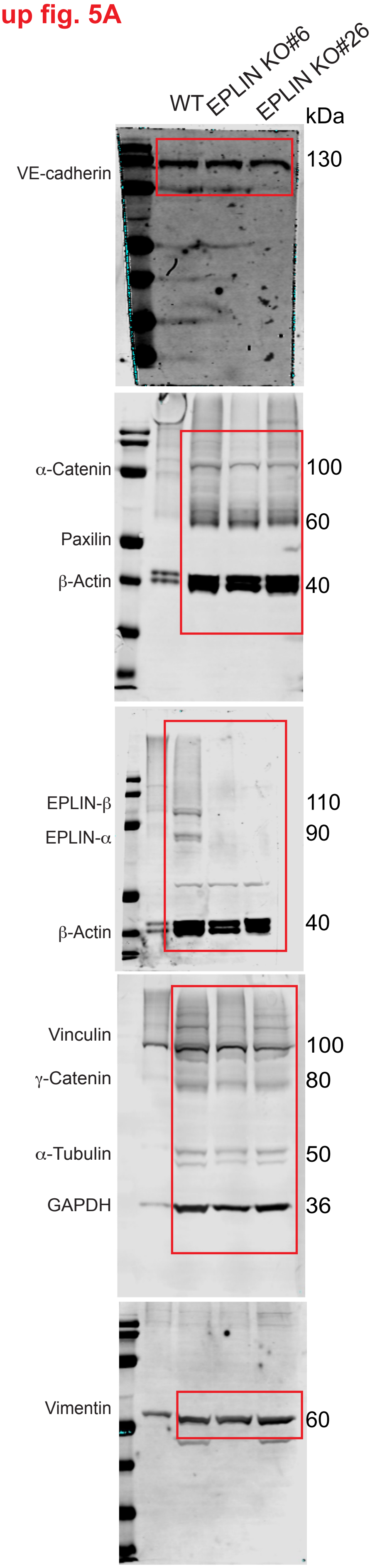


Supplementary Figure 7. Full immunoblots The area of the original blots shown in related supplementary figures are framed in red
