# Supplementary material for: A specific role for endothelial EPLIN-isoform-regulated actin dynamics in neutrophil transmigration
Source: Sci Rep. 2025 May 5;15:15698. doi: 10.1038/s41598-025-98192-9 (PMC12053001; doi:10.1038/s41598-025-98192-9)
Supplement: Supplementary file 4 — Supplementary Information 2. [file 41598_2025_98192_MOESM4_ESM.docx]

**Legends for Supplementary Videos**

**Movie 1.** Dynamics of EPLIN-isoforms during trans-endothelial migration of leukocytes through TNF-α activated confluent HUVEC cultures (10^5^ cells/cm^2^) moderately expressing either EPLIN-α-EGFP (upper panel) or EPLIN-β-EGFP (lower panel). After firm adhesion of leukocytes to endothelium EPLIN-α-EGFP recruits to gaps, forming a ring-like structure, that subsequently shortens until the leukocytes have passed the endothelium. Afterwards, EPLIN-α-EGFP appears sequentially - which is in agreement with the formation of membrane protrusions to close the gaps. EPLIN-β-positive ring was also recognized during TEM, which remains at the transmigrating leucocyte. However, we did not observe membrane protrusion like signals.

**Movie 2.** Analyses of the Arp2/3 complex dynamics during TEM of leukocytes though activated and EPFP-p20 (a subunit of the Arp2/3 complex) expressing confluent HUVEC cultures (10^5^ cells/cm^2^). The Arp2/3 complex positive JAIL appear in TNF-α activated cells at cell junctions as indicated by EGFP-p20 (arrows). Quickly after attachment, small EGFP-p20 positive structures appear while leukocytes pass the endothelium. Importantly, after leukocytes have passed the endothelium, EGFP-p20 positive protrusions appear at multiple sites to close the gap.
